# Resistance-CONKAT-seq Guided Discovery of a ClpP Active Natural Product from a Soil Metagenome

**DOI:** 10.1021/acschembio.6c00347

**Published:** 2026-06-29

**Authors:** Jingbo Kan, Adrian Morales-Amador, Yozen Hernandez, Ján Burian, Melinda A. Ternei, Sean F. Brady

**Affiliations:** Laboratory of Genetically Encoded Small Molecules, 5929 The Rockefeller University, 1230 York Avenue, New York, New York 10065, United States

## Abstract

The discovery of natural products with specific molecular targets from metagenomes remains challenging. To address this limitation, we developed resistance-CONKAT-seq (resistance co-occurrence network analysis of targeted sequences) which links metagenomic BGCs (biosynthetic gene clusters) to potential modes of action through the identification of colocalized molecular target-based resistance genes. Applying this approach to a soil metagenomic library, we identified the uncharacterized metagenomic azetidopyrroline (MTA) BGC associated with a potential *clpP* self-resistance gene. Genetic engineering and heterologous expression of the MTA BGC led to the discovery of metaze A and B, which are structurally related azetidopyrroline- and bicyclocarbamate-based natural products, respectively. Metaze B inhibited *Mycobacterium tuberculosis* caseinolytic protease proteolytic subunit (ClpP) with an IC_50_ of 1.35 μM. This study expands the chemical diversity of natural product ClpP inhibitors and further demonstrates the applicability of resistance-CONKAT-seq for target-guided discovery of natural products with specific modes of action from complex metagenomes.

## Graphical abstract

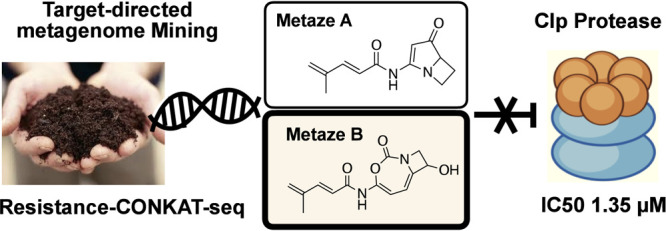

The study of bacterial natural products has been the key source of antibiotic development leads, inspiring the majority of clinically used antibacterial agents.^1,2^ Even with this rich history most of the biosynthetic potential of environmental microbiomes remains untapped, as the majority of bacteria remain uncultured so their natural products escaped previous laboratory-culture based discovery efforts.^3–5^ While these uncultured organisms harbor a large collection of small molecule biosynthetic gene clusters (BGCs), their products remain inaccessible without the development of additional discovery strategies. Sequence-based metagenomics has emerged as a powerful approach to access this hidden reservoir of compounds through environmental DNA cloning, BGC identification, and heterologous expression of target BGCs. One key challenge of this process is determining which BGCs to prioritize for downstream analysis.

The discovery of biomedically relevant bacterial natural products has most often relied on random screening of culture broth extracts. A random screening approach with metagenomics remains challenging due to the labor-intensive nature of the process. A more efficient strategy would be to prioritize BGCs based on their likelihood of encoding compounds with biomedically promising modes of action (MOAs). The colocalization of BGCs with genes predicted to encode resistant variants of a natural product’s molecular target (i.e., self-resistance genes) has been increasingly used to identify potentially interesting BGCs in sequenced bacterial genomes.^6–10^ In fact, bioinformatic tools have been developed to facilitate the automated analysis of fully sequenced genomes for BGCs colocalized with potential self-resistance genes to prioritize targets for natural product discovery.^11–13^ While this molecular target-guided approach is straightforward to apply when examining organisms with completely sequenced genomes, its application to metagenomes has been limited due to high sequencing costs and the typically short length of assembled DNA fragments, which are often insufficient to capture full-length BGCs. To address this limitation, we recently developed resistance-CONKAT-seq (resistance co-occurrence network analysis of targeted sequences), a high-throughput metagenomic mining approach that links BGCs to putative mechanisms of action through identification of biosynthetic motifs colocalized with genes predicted to confer self-resistance.^14^ CONKAT-seq was originally developed to identify metagenomic BGCs by tracking the co-occurrence of PCR-amplified biosynthetic domain sequences across arrayed (meta)­genomic libraries.^15^ Resistance-CONKAT-seq (Figure 1a) extends this approach by linking potential self-resistance genes to biosynthetic gene networks by simultaneously tracking amplified sequences of candidate self-resistance genes. This enables MOA prediction directly from metagenomic libraries without complete genome sequencing and assembly, which renders target-guided discovery both practical and scalable for metagenomic library screening.

**Figure 1:**
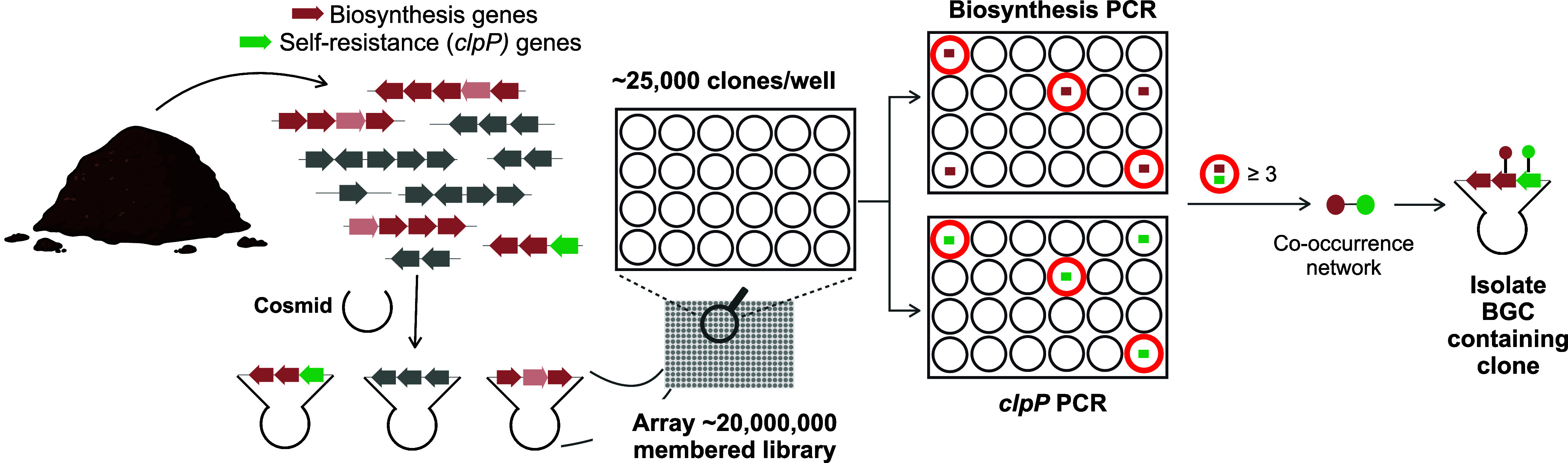
Schematic of resistance-CONKAT-seq. CONKAT-seq leverages PCR amplification of conserved biosynthetic gene fragments and the partitioned nature of an arrayed metagenomic library to generate networks of biosynthetic motifs that are associated with unique BGCs. Resistance-CONKAT-seq enables the identification of BGCs encoding metabolites with specific protein targets in complex metagenomes by tracking the colocalization of self-resistance genes with biosynthetic gene networks. BGCs are represented by red colored arrows. Resistance genes are represented by green colored arrows. Each node in the network represents a gene and each edge indicates a co-occurrence event between two genes.

Here, we applied resistance-CONKAT-seq to identify natural products targeting caseinolytic protease P (ClpP), a critical bacterial protease involved in the viability and pathogenesis of multiple pathogens^16–19^ for which no therapeutics have yet been developed. Resistance-CONKAT-seq screening of a soil metagenomic cosmid library led to the discovery of a cryptic nonribosomal peptide synthetase (NRPS) BGC that colocalized with a *clpP* gene. Heterologous expression of this BGC yielded two previously uncharacterized compounds, metaze A and metaze B, which belong to the azetidine-containing alkaloid family^20^ but feature a distinct fatty acid tail that expands the structural diversity of this class. Metaze B exhibited potent inhibition of the *Mycobacterium tuberculosis* (Mtb) ClpP1/P2:C1 complex (IC_50_ = 1.35 μM).

NRPSs and polyketide synthases (PKSs) are responsible for producing two major classes of natural products, nonribosomal peptides and polyketides, which together account for a large proportion of biomedically relevant compounds.^2,21^ Both NRPS and PKS biosynthesis proceed through the repetitive use of conserved domain sets (modules) to couple simple building blocks into larger polymers. This organization allows the amplification of multiple biosynthetic domains from a BGC using a single set of degenerate primers. Due to their repetitive, modular nature and long history of biomedical productivity, NRPS and PKS BGCs represent ideal targets for CONKAT-seq. To identify *clpP* associated BGCs, we performed resistance-CONKAT-seq on an archived soil metagenomic DNA cosmid library (OR13) containing over 2 × 10^7^ unique clones. During its construction, this library was arrayed in 384-well plates as pools of approximately 25 000 unique clones per well (Table S1). We first used well-barcoded degenerate primers specific for NRPS adenylation (A-) or PKS ketosynthase (KS-) domains to map NRPS and PKS BGCs across the OR13 library. We then used well-barcoded *clpP* degenerate primers to amplify *clpP* genes across the library. In our initial report of resistance-CONKAT-seq, amplicon sequencing was performed using next-generation Illumina platforms.^22^ Substantial improvements in read accuracy and reductions in per-sample costs have made long-read (third-generation) sequencing technologies increasingly attractive for large amplicon-based sequencing studies.^23–25^ The extended read lengths provided by long-read platforms are particularly advantageous for resistance-CONKAT-seq, as short amplicons generated using Illumina methods cannot always resolve highly similar gene variants, potentially leading to false-positive connections within resistance networks. In light of these advantages, we transitioned to Oxford Nanopore for all A-domain and *clpP* amplicon sequencing performed in this study. We found that this transition reduced amplicon sequencing costs by 2-fold, making resistance-CONKAT-seq even more economical and scalable. Sequences were demultiplexed based on the well-specific barcodes and then domain networks containing biosynthetic and *clpP* domains were identified using the resistance-CONKAT-seq algorithm. This analysis identified one *clpP* associated A-domain network (Figure S1) and no KS-containing networks with associated *clpP* genes. This result was not unexpected, as BGCs encoding a specific MOA are likely to be rare within individual metagenomes. Resistance-CONKAT-seq was used to aid in the discovery of these rare BGCs.

The cosmid associated with the AD*-clpP* containing network was recovered from the library, sequenced and annotated. This cosmid contains a fragment of the saccharochelin^26^ BGC on one end with four A-domain nodes (left circle in the network, Figure 2a), and a *clpP*-adjacent NRPS BGC with multiple tailoring enzymes on the other with two A-domain nodes (right circle in the network; Figure 2a). The *clpP*-adjacent cluster’s overall organization closely resembles that of the BGC that encodes azetidomonamide A and B. Within azetidomonamide A, the bicyclocarbamate moiety provides ClpP inhibitor activity^27^ (Figure 2b). The MTA BGC differs by the presence of an additional transcriptional regulator at the cluster edge (*mtaA*), an extra acyl-CoA dehydrogenase gene (*mtaJ*), and a relocated *clpP* gene (*mtaL*) (Figure 2b, c). The individual genes within the *clpP*-adjacent cluster show high similarity to an uncharacterized BGC from a *Saccharothrix* species (Figure 2c). The similarity to the known ClpP inhibitor BGC and the presence of the unique tailoring enzyme suggested that this cluster would encode a new ClpP-targeting natural product within the azetidopyrroline family. We named this cluster the MTA BGC (metagenomic azetidopyrroline).

**Figure 2:**
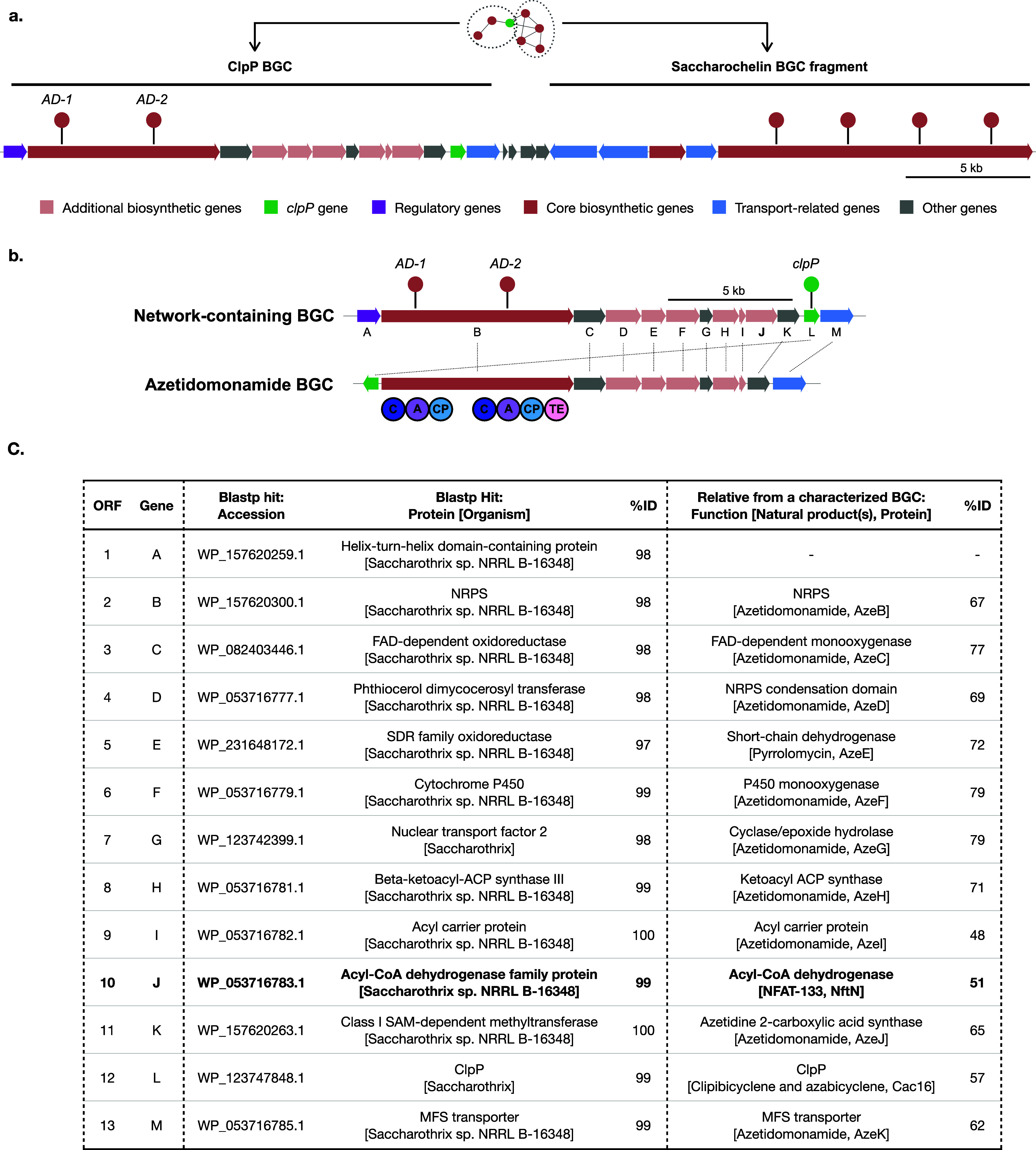
Discovery of the MTA BGC. (a) Depiction of the AD (A-domain)-*clpP* network that led to the discovery of the MTA BGC from the OR13 library is shown on top of the annotated cosmid from which this network arose. (b) A comparison of the *clpP* gene containing BGC (MTA BGC) with the azetidomonamide BGC from *Pseudomonas aeruginosa* is shown. [Legend: C, Condensation domain; A, Adenylation domain; CP, carrier protein; TE, Thioesterase domain.] (c) Information for each predicted gene in the MTA BGC. The additional biosynthetic gene (*mtaJ*) compared to the azetidomonamide BGC is highlighted in bold. Identity (%) represents the percentage of protein sequence identity.

To access the metabolite(s) encoded by the MTA BGC, we used transformation assisted recombination (TAR) in yeast to transfer the BGC to an *E. coli*-*Streptomyces*-yeast bacterial shuttle vector (pTAR-lys) and then conjugated this construct into *Streptomyces albus* J1074 for heterologous expression studies. Compared to the *S. albus* empty vector control culture, a single clone specific metabolite *(* *m*/*z* = 219*)* with low signal intensity was detected in the methanol extract of dried *S. albus* cultures harboring the MTA BGC (Figure 3a). The MTA BGC appears to be a single operon, or at least a set of genes all transcribed in the same direction. Therefore, in an effort to increase production of the encoded metabolite we used *mi*CASTAR,^28^ a TAR/CRISPR-based BGC refactoring protocol, to introduce a strong *streptomyces*-active promoter in front of the first gene (*mtaA)* in this predicted operon. In cultures of *S. albus* transformed with this engineered construct, we observed an approximately 20-fold increase in the signal of the *m*/*z* 219 feature (Figure 3b). An optimization study of extraction methods found that XAD-7 HP resin recovered the highest amount of this metabolite (Figure 3c). The compound was isolated from large-scale cultures of the promoter engineered construct using XAD-7 HP resin and the structure was elucidated using a combination of MS and NMR data (see Figure 3d, as well as Figure S1–7 and Table S4). The deduced structure contains a bicyclic azetidopyrroline core that is N-acylated with a 4-methylpenta-2,4-dienoic acid moiety. We gave this natural product the trivial name metaze A (metagenomic azetidopyrroline). Metaze A differs from azetidomonamide B by the presence of the *4-methylpenta-2,4-dienoic acid* in place of a crotonic acid. The additional acyl-CoA dehydrogenase (MtaJ) in the MTA BGC likely explains this structural difference (Figure S8).

**Figure 3:**
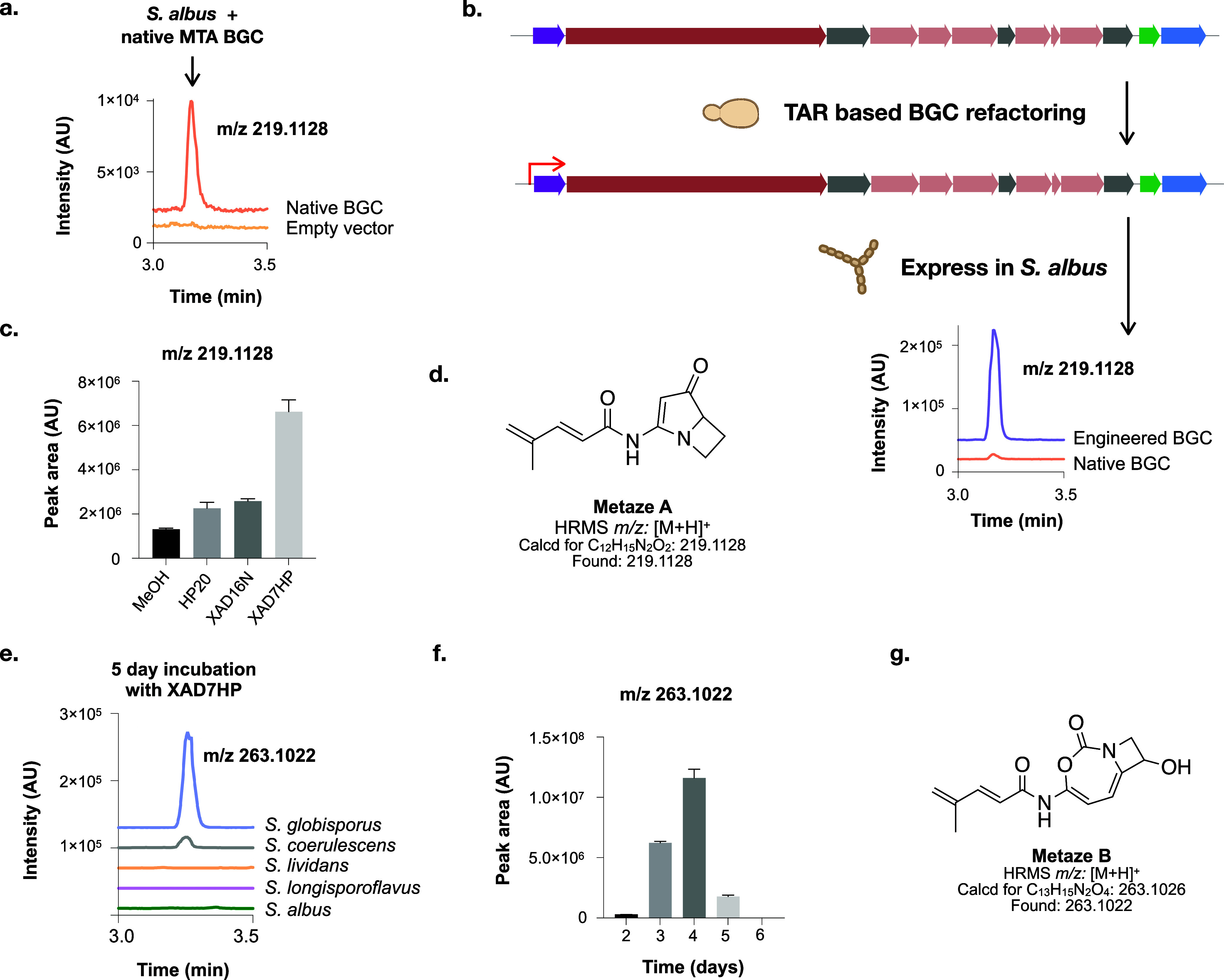
Discovery of Metaze A and B. (a) HPLC-MS extracted ion chromatograms (*m*/*z* 219.1128) of culture broth extracts from *S. albus* transformed with an empty vector and the vector containing the native MTA BGC. Each curve represents data from one of two replicates. (b) HPLC-MS extracted ion chromatograms (*m*/*z* 219.1128) of culture broth extracts from *S. albus* transformed with the native MTA BGC and the promoter-engineered MTA BGC. Each curve represents data from one of two replicates. (c) Peak areas of HPLC-MS extracted ion chromatograms (*m*/*z* 219.1128) of culture broth extracts obtained using different extraction methods. Data are presented as mean ± SEM (*n* = 3). (d) HRMS data and chemical structure of metaze A. (e) HPLC-MS extracted ion chromatograms (*m*/*z* 263.1022) of culture broth extracts from different *Streptomyces* hosts transformed with the promoter-engineered MTA BGC. Each curve represents data from one of two replicates. (f) Peak areas of HPLC-MS extracted ion chromatograms (*m*/*z* 263.1022) of culture extracts from *S. globisporus* transformed with the promoter-engineered MTA BGC collected at different time points. Data are presented as mean ± SEM (*n* = 3). (g) HRMS data and chemical structure of metaze B.

Azetidopyrroline BGCs have been reported to produce pairs of closely related metabolites, one featuring an azetidopyrroline scaffold (e.g., metaze A) and the other containing a bicyclocarbamate ring system.^20,29^ The bicyclocarbamate structure is the direct product of the BGC but is unstable and spontaneously converts to the azetidopyrroline scaffold. We reasoned that the MTA BGC could also encode a bicyclocarbamate structure that was rapidly converted to metaze A. We searched for the predicted mass of the bicyclocarbamate analog of metaze A (*m*/*z* = 263.1026 [M–H]^+^) in the crude extract from which we isolated metaze A and observed a very weak signal (e^–3^). In an effort to identify heterologous expression conditions that would yield more of this metabolite, we both shuttled the promoter re-engineered metaze A construct into other *Streptomyces* hosts and explored shorter culture times. The highest intensity *m*/*z* 263 peak was observed in extracts from cultures of *Streptomyces globisporus* transformed with the re-engineered metaze A construct (Figure 3e). This signal peaked at 4 days and disappeared over time (Figure 3f). We were able to purify the *m*/*z* 263 compound from 4-day old recombinant *S. globisporus* cultures and solve its structure using HRMS and NMR (see Figure 3g, as well as Figures S9–S16 and Table S5). As predicted, this compound was the bicyclocarbamate analog of metaze A.

While azetidopyrrolines have no reported biological activity, the bicyclocarbamate-containing compound clipibicyclene has been shown to inhibit the ClpP protease.^30^ To test the effects of metaze A and B on ClpP, we performed an in vitro fluorescence-based protease assay using the Mtb Clp protease system, which consists of the ClpP1/P2 protease complex and the substrate unfoldase ClpC1. Since the AMC-based substrates typically used with the Mtb ClpP1/P2 complex alone are quenched by the bicyclocarbamate ring,^30^ we used the Mtb ClpP1/P2:C1 complex with FITC-casein as the substrate, which is not affected by metaze A or B. Metaze A did not exhibit any inhibitory effect at the highest concentration we tested (100 μM). However, metaze B inhibited ClpP activity in a concentration-dependent manner (Figure 4a) with an IC_50_ of 1.35 μM (Figure 4b). The analysis by intact protein liquid chromatography–mass spectrometry (LC-MS) showed a mass increase of ∼262 Da for both Mtb ClpP1 and ClpP2 (Figure S17a), suggesting that metaze B covalently modifies Mtb ClpP (Figure S17b). Neither metaze A nor B showed inhibitory activity against any of the organisms we tested including bacteria, fungi, and human cells (Table S6). This is consistent with the lack of growth inhibition observed for other congeners within this natural product family and may be due to the instability of the bicyclocarbamate ring.^20,29,30^

**Figure 4:**
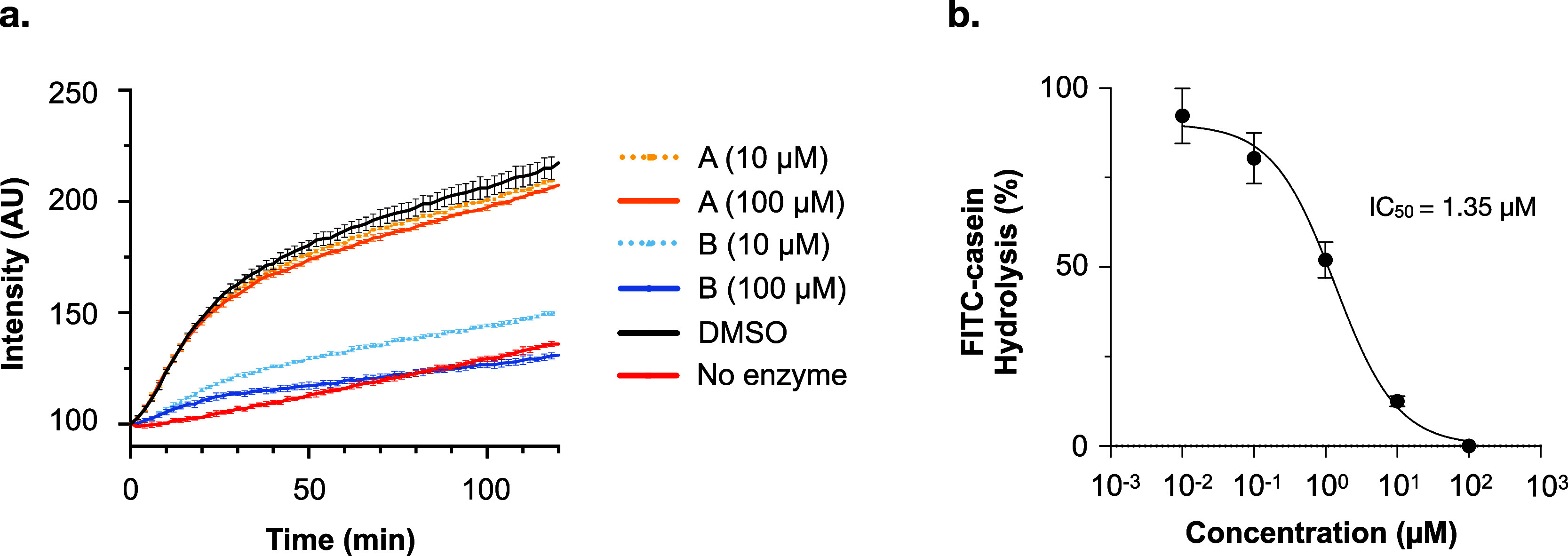
Metaze B inhibits Mtb ClpP. (a) Mtb ClpP1/P2:C1 protease activity assay performed using FITC-casein (fluorescein isothiocyanate labeled casein) as the substrate. (b) IC_50_ curve of metaze B inhibition of Mtb ClpP1/P2:C1 protease activity. Error bars indicate the standard deviation of three biological replicates.

Earlier reports found that azetidomonamide BGCs were widespread among cultured sequenced bacteria.^30^ Our discovery of metaze A and B suggests that this natural product family is likely also common among uncultured bacteria. The instability of the bicyclocarbamate ring found in metaze B and related molecules likely contributes to their underrepresentation in natural product libraries, as these electrophilic warheads are prone to degradation during standard fermentation and extraction protocols. In this study, metaze B production levels declined after 4 days of incubation, while addition of resin to the culture broth to rapidly sequester the unstable metabolite improved recovery. This suggests that specific strategies may be required to isolate and identify these unstable, yet bioactive, compounds. The detection of bicyclocarbamate structures in future screenings of BGCs predicted to encode this warhead would likely benefit from the implementation of similar fermentation and extraction conditions, enabling a more comprehensive investigation of the biosynthesis and bioactivities associated with this class of compounds.

Our discovery of metaze B with ClpP inhibitory activity highlights the potential of resistance-CONKAT-seq as a platform for identifying natural products with desired MOAs from complex soil metagenomes. Although ClpP was used as the molecular target in this study, resistance-CONKAT-seq could be extended to other resistance MOAs by PCR targeting other potential self-resistance gene classes. We believe that successfully transitioning from Illumina technology to Oxford Nanopore sequencing of amplicons not only makes resistance-CONKAT-seq more economical and scalable, but also more user-friendly. With the ever-decreasing cost of next-generation sequencing, resistance-CONKAT-seq has the potential to serve as a broadly applicable tool for prioritizing the discovery of valuable natural products with specific desired molecular targets from complex metagenomes.

## Materials and Methods

### Gene amplification and sequencing

The OR13 soil metagenomic library was constructed previously and archived as solutions of cosmid DNA pools across two 384-well plates and matching *E. coli* clone glycerol stocks. Barcoded degenerate primers targeting *clpP* (Deg-ClpP-F2/Deg-ClpP-R2), A-domain (AD3-F/AD3-R), and KS-domain (KS3-F/KS3-R) sequences were used to generate amplicons from each library well in 10 μL PCR reactions. The reaction mix included 5 μL of 2× FailSafe PCR PreMix G (Biosearch Technologies), 0.2 μL of each barcoded primer (100 μM), 1 μL of template cosmid DNA (20 ng/μL), 0.2 μL of rTaq polymerase (Bulldog Bio), and 3.4 μL of nuclease-free H_2_O. PCR of *clpP* was performed using the following conditions: 95 °C for 5 min, followed by 35 cycles of 95 °C for 30 s, 55 °C for 30 s, and 72 °C for 30 s with a final extension at 72 °C for 5 min. PCR of A-domain sequences was performed using the following conditions: 95 °C for 5 min, followed by 35 cycles of 95 °C for 30 s, 56.3 °C for 30 s, and 72 °C for 40 s with a final extension at 72 °C for 5 min. PCR of KS-domain sequences was performed using the following conditions: 95 °C for 5 min, followed by 35 cycles of 95 °C for 30 s, 60 °C for 30 s, and 72 °C for 40 s with a final extension at 72 °C for 5 min. Amplicons from each of the PCRs across the wells for a library plate were separately pooled, gel-purified, and then were used to seed a second-round PCR to add plate specific barcodes. This second-round PCR was performed using the following conditions: 95 °C for 5 min, followed by 6 cycles of 95 °C for 30 s, 70 °C for 30 s, and 72 °C for 45 s with a final extension at 72 °C for 5 min. The PCR products of KS-domain amplicons with barcodes were purified using Agencourt AMPure XP magnetic beads (Beckman Coulter) and quantified using a 2200 TapeStation (Agilent Technologies). Final KS-Amplicons were pooled equimolarly and sequenced at a final concentration of 10 nM on an Illumina NextSeq platform. The PCR products of *clpP* and A-domain amplicons with barcodes were ligated with sequencing adapters using Oxford Nanopore’s Ligation Sequencing kit (SQK-LSK114) as per manufacturer’s instructions, purified with Agencourt AMPure XP magnetic beads (Beckman Coulter), quantified by Qubit (Invitrogen), and pooled equimolarly to a final concentration of 20 fmol. The sample was then loaded into a single PromethION flow cell (FLO-PRO114M) which was sequenced to exhaustion using a P2 Solo.

### Resistance-CONKAT-seq Analysis

The resistance-CONKAT-seq analysis was performed using the previously described pipeline^22^ with minor adjustments. Briefly, operational taxonomic units (OTUs) were clustered at 90% rather than 95% to account for the higher expected error rate of Nanopore reads. To identify *clpP* genes associated with AD/KS domain network clusters, *clpP* OTUs were analyzed using CONKAT-seq^31,32^ together with AD/KS OTUs. OTUs sharing at least three common wells were grouped into the same network. Networks were visualized in Cytoscape^33^ and candidates selected if they contained at least one *clpP* OTU and at least one AD/KS domain OTU. To eliminate possible false positive nodes, each OTU was subjected to taxonomic analysis using NCBI BLAST and OTUs belonging to different phyla than the *clpP* OTU were removed from the networks.

### Isolation of Metaze A

Spores of *Streptomyces albus* J1074 carrying the refactored MTA BGC were inoculated into 5 mL of trypticase soy broth (Oxoid) and incubated at 30 °C with shaking at 200 rpm for 48 h to generate a starter culture. A 100 μL aliquot of the starter culture was then added into five 2.8 L baffled flasks, each containing 1 L of R5a medium and 25 g of Amberlite XAD7HP resin (Sigma–Aldrich), followed by incubation at 30 °C and 200 rpm for 7 days. The resin was collected and extracted three times with 1 L of methanol. The methanol extracts were combined, concentrated to 250 mL using a rotary evaporator and adjusted to 500 mL of 90% methanol. This solution was extracted twice with an equal volume of hexane (1:1 v/v). The methanol phase was diluted with water to a final methanol concentration of 60%, then extracted twice with chloroform (1:1 v/v). The combined chloroform extracts were then dried using a rotary evaporator and resuspended in 10 mL of methanol. The resulting sample was passed through a 50 g C18 RediSep Rf column and separated by reversed-phase chromatography using a linear gradient of 5%–100% H_2_O/acetonitrile containing 0.01% formic acid over the course of 30 min. Fractions containing the target compound were further purified by semipreparative high-performance liquid chromatography (HPLC) using an XBridge C18 130 Å column (150 mm × 10 mm, 5 μm, Waters). Solvent A was water containing 0.1% formic acid, and solvent B was acetonitrile containing 0.1% formic acid. The purification was performed using a linear gradient of 5%–40% B from 0 to 40 min, 40–100% B from 40 to 50 min, 100–5% B from 50 to 52 min, 5% B from 52 to 60 min, at a flow rate of 3 mL/min.

### Isolation of Metaze B

Culturing and bulk extraction of metaze B were performed as described for metaze A. For the isolation of metaze B, the combined chloroform extracts were passed through a 50 g C18 RediSep Rf column and separated by reversed-phase chromatography using a linear gradient of 5–100% H_2_O/acetonitrile containing 0.1% formic acid over the course of 30 min. Fractions containing the target compound were loaded onto a hand-filled chromatography column containing 50 g of C18 silica gel (60 Å, 40–63 μm, Sorbtech). The loaded resin was washed in turn with 100 mL of water and 20 mL of 50% methanol, and the pure metaze B fraction was eluted by 20 mL of methanol.

### *In vitro* ClpP protease assays and IC_50_ measurement

The *in vitro* Mtb ClpP protease assays were performed as previously described.^22^ Briefly, ClpP1, ClpP2, and ClpC1 (each at 1 μM, monomer) were combined in the assay buffer from the Mtb ClpP Protease Assay Kit (ProFoldin) supplemented with 8 mM MgCl_2_ and 2 mM ATP. Test compounds were preincubated with the mixed buffer containing enzymes for 5 min before adding 0.3 μL of 200 μM FITC-casein (AAT Bioquest) as the fluorogenic substrate. Fluorescence was measured at 37 °C on an Infinite M200 Pro plate reader (TECAN) using excitation/emission wavelengths of 480/520 nm. The IC_50_ of metaze B against Mtb ClpP1/P2:C1 was determined using five concentrations (0.01, 0.1, 1, 10, and 100 μM) of the compound. FITC-casein hydrolysis rate was calculated as the fluorescence change rate over 60 min. The inhibition percentage (%) was calculated as follows: the hydrolysis rate of each sample minus the hydrolysis rate of the no-enzyme control divided by the hydrolysis rate of the DMSO control. All assays were performed in triplicate and the data plotted using GraphPad Prism 10.

## Supplementary Material



## Data Availability

Source data are provided with this study. The assembled sequencing data has been submitted to the NCBI GenBank database, under Accession No. PX841131.

## Notes

Supplementary methods, soil sample characteristics, sequencing details, primers, strains and constructs, and NMR spectra for all compounds (PDF)
