# Supplementary material for: Resistance-CONKAT-seq Guided Discovery of a ClpP Active Natural Product from a Soil Metagenome
Source: ACS Chem Biol. 2026 Jun 29;21(7):1615–22. doi: 10.1021/acschembio.6c00347 (PMC13386464; doi:10.1021/acschembio.6c00347)
Supplement: Supplementary file 1 [file cb6c00347_si_001.pdf]

Supplementary information for:

Title: Resistance-CONKAT-seq guided discovery of a ClpP active natural product from a soil metagenome.

Authors: Jingbo Kan, Adrian Morales-Amador, Yozen Hernandez, Ján Burian, Melinda A. Ternei, Sean F. Brady*

Author affiliation: Laboratory of Genetically Encoded Small Molecules, The Rockefeller University, 1230 York Avenue, New York, NY 10065, USA

***Corresponding Author:** Sean F. Brady

Contact: Laboratory of Genetically Encoded Small Molecules
The Rockefeller University
1230 York Avenue
New York, NY 10065
Phone: 212-327-8280
Fax: 212-327-8281
Email: sbrady@rockefeller.edu

Supplementary methods

Recovery of BGC encoding clones from the soil cosmid library

The library well containing the putative BGC-harboring clone was identified based on the well barcode associated with the resistance-CONKAT-seq network. Specific forward and reverse primer pairs were designed to amplify the A-domain (OR13-AD-F/ OR13-AD-R) and *clpP* genes (OR13-ClpP-F/ OR13-ClpP-F-R) within the target network, respectively. Clones were isolated using the serial dilution PCR method as previously described[1]. The isolated clone was cultured, the cosmid DNA was purified using the Monarch® Plasmid Miniprep Kit (NEB), and the cosmid was sequenced with the Nextera XT protocol on a MiSeq instrument (Illumina). Finally, the clone sequence was assembled and annotated using antiSMASH (bacterial version) and NCBI BLAST based on predicted protein functions and homology.

Heterologous expression

The cosmid harboring the MTA BGC was integrated into a custom shuttle capture vector (pTAR-lys) containing *ϕ*C31 integration elements for genomic insertion into *Streptomyces* hosts (*S. albus*, *S. globisporus*, *S. coerulescens*, *S. lividans*, and *S. longisporoflavus*) through transformation-associated recombination (TAR) in yeast as previously described[2]. Briefly, pTAR-lys was modified to include approximately 500 bp homology arms corresponding to the terminal regions of the target cosmid, the capture vector linearized with *PmeI* (NEB), the target cosmid linearized with *DraI* (NEB), and the digested DNA fragments co-transformed into *Saccharomyces cerevisiae* BY4727 using the standard spheroplast method[3] to yield yeast colonies harboring the cosmid DNA inserted into the capture vector. The assembled construct was confirmed by sequencing, transformed into *E. coli* S17 and conjugated into *Streptomyces* hosts. Spore stocks were generated from PCR-validated conjugants and used to inoculate 5 mL of trypticase soy broth (Oxoid). Inoculated cultures were then incubated at 30 °C with shaking at 200 rpm for 48 hours to promote mycelial growth. Then, a 0.05 mL aliquot of the starter culture was transferred into 50 mL of R5a medium (100 g/L of sucrose, 5 g/L of yeast extract, 10.12 g/L of $MgCl_2·6H_2O$, 0.25 g/L of K_2SO_4, 0.1 g/L of casamino acids, 21 g/L of MOPS, 10 g/L of *D*-glucose, 2 g/L of NaOH, 5.88 mg/L of $CaCl_2$, 80 μg/L of $ZnCl_2$, 400 μg/L of $FeCl_3·6H_2O$, 20 μg/L of $MnCl_2$, 20 μg/L of $CuCl_2$, 20 μg/L of $Na_2B_4O_7·10H_2O$, 20 μg/L of $(NH_4)_6Mo_7O_{24}·4H_2O$, pH 6.8) in 125 mL baffled flasks and incubated at 30 °C and 200 rpm for 10 days.

Biosynthetic gene cluster refactoring

The MTA BGC was refactored using miCASTAR as previously described[4]. In brief, single guide RNA (sgRNA) primers targeting promoter cassette insertion sites were designed and synthesized using the EnGen sgRNA synthesis Kit (NEB). Synthesized sgRNAs were purified with the RNA Clean & Concentrator Kit (Zymo Research) and the MTA BGC containing TAR vector was digested with Cas9 Nuclease (NEB) in the presence of the sgRNAs at 37 °C overnight, and the linearized DNA was purified using the ZR Research BAC DNA Miniprep Kit (Zymo Research). A promoter cassette containing a prototrophic marker, a strong *Streptomyces* promoter (SNP11), and flanking homology arms was amplified by PCR using OR12_SNP11_UP and OR12_SNP11_DN. The amplified promoter cassette and Cas9-digested MTA BGC were then co-transformed into *Saccharomyces cerevisiae* BY4727 using the standard spheroplast method as described above. Refactored clones were validated by sequencing, transformed into *E. coli* S17 cells, and conjugated into *Streptomyces albus* J1074 for heterologous expression as described above.

Nuclear magnetic resonance (NMR) analysis

NMR spectra were acquired on a Bruker Avance NEO equipped with a TCI cryogenic probe (The Rockefeller University) operating at 600 MHz for ^1H and 150 MHz for ^{13}C nuclei in CD_3OD for Metaze A and B. The sample temperature was set at 298.2 K. The chemical shifts are reported in ppm using the residual solvent peak as internal reference, set at 3.31 ppm for ^1H and 49.0 ppm for ^{13}C. All NMR experiments were run using standard pulse sequences in the phase sensitive mode. The spectra were analyzed and visualized using TopSpin (3.6.0) and MestReNova (14.3.0). Multiplicity and coupling constants are quoted where possible (br: broad, d: doublet, dd: doublet of doublets, m: multiplet, q: quartet, s: singlet, t: triplet).

High resolution mass spectrometry analysis

Electrospray ionization coupled with liquid chromatography–high resolution mass spectrometry (LC-HRMS) data were acquired using a SCIEX Exion HPLC system coupled to an X500R QTOF mass spectrometer (The

Rockefeller University). The system was equipped with a Phenomenex Kinetex PC C18 100Å column (50 mm x 2.1 mm, 2.6 μm) and operated with SCIEX OS v2.1 software. The following chromatographic conditions were used for LC-HRMS: 5% B to 0.5 min, 5-95% B from 0.5 to 6.5 min, 95% B from 6.5 to 8.5 min, finally 95% B to 5% B from 8.5 to 8.6 min and maintained to 10 min (A: water, B: acetonitrile, both containing 0.1% of formic acid). In positive electrospray ionization mode (+ESI), full HRMS spectra were acquired in the range m/z 100-1500, applying a declustering potential of 80 V, collision energy of 5 V, source temperature of 500 °C and a spray voltage of 5500 V. A maximum of 7 candidate ions from every Full HRMS event were subjected to Q2–MS/MS experiments in the range of m/z 50-1500, applying a collision energy of 35 ± 10 V. Full HRMS and the most intense MS/MS spectra were analyzed with MestReNova (14.3.0) and MZmine (3.6.0).

Metabolomics analysis and identification of unique features

For the identification of expressed molecules, the untargeted (data-dependent) LC-HRMS datasets were subjected to metabolomics analysis. The raw data were converted to mzXML files and processed in MZmine 3.6.0 following these steps: Mass detection was carried out using the centroid detector with a noise level of 3000. Chromatograms were generated with the ADAP Chromatographic Builder tool (minimum consecutive scans = 5; minimum intensity = 300; minimum absolute height = 3000; *m/z* tolerance = 10 ppm). Chromatographic deconvolution of the obtained EICs was achieved using the Local Minimum Resolver tool (chromatographic threshold = 85%; minimum search range RT = 0.5 min; minimum relative height = 1%; minimum absolute height = 3000; minimum ratio of peak top/edge = 1.3; minimum scans = 3). The resulting feature list was curated by eliminating redundant isotopes corresponding to the same chemical entities through the 13C isotope filter tool (*m/z* tolerance = 10 ppm; RT tolerance = 0.5 min; maximum charge = 1). Subsequently, all chromatograms from every feature across the extracts were aligned with the Join Aligner algorithm (*m/z* tolerance = 10 ppm; weight for *m/z* = 20; RT tolerance = 0.5 min; weight for RT = 10). Alignment gaps were then filled using the Peak Finder (intensity tolerance = 0.05%; *m/z* tolerance = 10 ppm; RT tolerance = 0.5 min). The final feature list was examined to identify ions associated with a single BGC, determined by their consistent presence in all the replicates of the culture for that specific clone.

Minimum inhibitory concentration (MIC) assays

Saturated cultures were prepared by inoculating glycerol stocks into the appropriate media and incubating under standard growth conditions: *M. smegmatis* mc^2 155 glycerol stock was inoculated into 5 mL of 7H9 liquid medium (0.94 g/200 mL of 7H9 powder, 50% glycerol, and 40% glucose, pH=7) followed by incubation at 37 °C with shaking at 200 rpm for 48 hours. *M. tuberculosis* mc^2 6206 glycerol stock was inoculated into 10 mL of defined 7H9 medium (0.94 g/200 mL of 7H9 powder, 50% glycerol, 20 mL/200 mL of ADC, 10 mg/mL of leucine, and 5.76 mg/mL of calcium pantothenate, pH=7) and incubated at 37 °C with 5% CO_2 for 14 days. *Candida albicans* ATCC 18804 glycerol stock was inoculated into 5 mL of YPD medium (BD Difco) and incubated at 30 °C with shaking at 200 rpm for 16 hours. Glycerol stocks of other bacterial strains were inoculated into 5 mL of LB medium and incubated at 37 °C with shaking at 200 rpm for 16 hours. Saturated cultures were diluted 1:5,000 into 10 mL of 7H9 medium for *M. smegmatis*, defined 7H9 medium for *M. tuberculosis*, YPD medium for *C. albicans*, and LB medium for other bacterial strains. Compound stock solutions were serially diluted 2-fold in a 96-well plate (ThermoFisher Scientific) containing the corresponding culture medium, and then an equal volume of diluted culture was added to each well and mixed by pipetting. Plates were incubated under organism-specific conditions (48 hours for *M. smegmatis*, 7 days for *M. tuberculosis*, and 16 hours for *C. albicans* and the other bacteria). The top and bottom plate rows contained empty growth medium to minimize edge effects. The final column lacked antibiotic and served as the control for cell viability. MIC values were determined by visual inspection as the lowest compound concentration that inhibited visible growth. All assays were performed in duplicate.

Human cell cytotoxicity assay

The cytotoxicity of metaze A/B was performed using the MTT (3-(4,5-Dimethyl-2-thiazolyl)-2,5-diphenyl-2H-tetrazolium bromide) assay. HEK293 cells were seeded at 5,000 cells/well in DMEM supplemented with 10% bovine serum and 1% glutamine, then incubated at 37 °C with 5% CO_2 for 24 hours. Serially diluted compounds were added and cells incubated for 48 hours. Medium was removed and 100 μL of MTT solution (0.5 mg/mL in DPBS) was added for 4 hours, followed by 100 μL of solubilization solution (40% DMF, 15% SDS, 2% acetic acid). After 1 hour at room temperature the absorbance at 570 nm was measured on an Infinite M200 Pro plate reader (TECAN). Paclitaxel and 0.2% DMSO were used as positive and negative controls, respectively.

Overexpression and purification of *Mtb* ClpP1/P2 and ClpC1

The expression and purification of Mtb ClpP1, ClpP2, and ClpC1 proteins were performed as previously described[1]. In brief, *clpP1* and *clpP2* were cloned with N-terminal 14×His-bdSUMO tags, and *clpC1* was cloned with an N-terminal 14×His tag. *Escherichia coli* BL21 (DE3) cells carrying the expression plasmids were grown in 1 L of LB medium at 37 °C with 200 rpm shaking to an OD_{600} of 0.8. The cultures were cooled to 4 °C for 20 minutes, induced with 0.5 mM isopropyl β-D-1-thiogalactopyranoside (IPTG) and incubated overnight at 16 °C with 200 rpm shaking. Cells were harvested by centrifugation (5,000 x g, 10 min) and lysed by sonication. The lysates were centrifuged (15,000 x g, 30 min) at 4 °C to remove cell debris, and the supernatant was applied to a 5 mL HiTrap chelating Ni^{2+} affinity column (GE Healthcare). The column was washed with approximately 50 column volumes of wash buffer (20 mM Tris, 250 mM NaCl, 30 mM imidazole, pH 8.0) and bound proteins were eluted with 25 mL of the same buffer supplemented with 300 mM imidazole. For ClpP1 and ClpP2, the bdSUMO tag was removed by incubation with 1 µg/mL bdSENP1 protease[5]. Protein elutions were concentrated to 5 mL using an Amicon Ultra centrifugal filter unit (100 kDa cutoff, Millipore), and Mtb ClpP1 and ClpP2 were further purified by size-exclusion chromatography (SEC) on a Superdex 200 10/300 GL column (GE Healthcare).

Intact protein LC-MS analysis

Metaze B (100 µM), Mtb ClpP1 (20 µM), and Mtb ClpP2 (20 µM) and the control sample without metaze B were respectively mixed in 100 µL of reaction buffer (20 mM Potassium phosphate, 0.1 mM KCl, 5% Glycerol) and incubated at 37°C for 2 hours. After the incubation, 2 µL of each sample was injected and separated using a MAbPac RP 4 µm 50 x 1 mm column (Thermo Fisher) coupled to an QE-HF (Thermo Fisher) using direct-loading. Analytes were eluted at 75uL/min using a gradient increasing from 2% A to 80% A (A: 0.1% formic acid, 0.02% TFA, B: Acetonitrile, 0.1% formic acid, 0.02% TFA) in 10 minutes. Data were recorded in one scan range (MS1-only at 45,000 res): 2000 - 4000 m/z. In-source CID at 50V. ES potential set at 3.6kV. Aux gas at 7. Sheet gas at 15. The mass spectrometer was calibrated prior to analysis. Data were analyzed using Xcalibur v.4.0.27.19 and UniDec GUI v.1.0.10.

Structural Elucidation:

Metaze A was detected during the analytical screening as a unique feature at *m/z* 219.1128 $[M+H]^+$, consistent with the molecular formula $C_{12}H_{14}N_2O_2$ (**Figure S1**). Detailed analysis of the ^1H, ^{13}C, and HSQC NMR data revealed the presence of three sp^2 methines, one deshielded sp^3 methine, one sp^2 methylene, one sp^3 methylene, one deshielded sp^3 methylene and one sp^3 methyl group. In addition, combined interpretation of the ^{13}C and ^1H-^{13}C HMBC spectra enabled the identification of two carbonyl carbons: one assigned to a heteroatom-substituted carbonyl (δ_{13C} 166.1 ppm), consistent with an amide or ester functionality, and another characteristic of a ketone group (δ_{13C} 209.0 ppm). Two non-protonated sp_2 carbons were also identified. The exocyclic methylene protons of CH_2-11 (δ_{13C} 126.1; δ_{1H} 5.47/5.46 ppm), showed heteronuclear long-range correlations with the non-protonated sp^2 carbon C-10 (δ_{13C} 141.8 ppm) and the methyl CH_3-12 (δ_{13C} 18.2), supporting the presence of a 2-substituted allyl moiety. This fragment was connected to CH-9 (δ_{13C} 148.2; δ_{1H} 7.47 ppm) through HMBC correlations from H-9 with C-10, togther with the HMBC correlations between CH-9, CH_2-11 and CH_3-12. CH-9 was further connected to CH-8 (δ_{13C} 120.9; δ_{1H} 6.20 ppm) by COSY analysis. Finally, both CH-8 and CH-9 showed clear HMBC correlations to the amide carbonyl C-7 (δ_{13C} 166.1). The large vicinal coupling constant between H-8 and H-9 ($^3J_{8H,9H}$ 15.5 Hz) establishes an *E* configuration for this double bond, allowing this substructure to be assigned as (*E*)-4-methylpenta-2,4-dienamide (**Table S4**).

The deshielded methine CH-2 (δ_{13C} 68.6; δ_{1H} 4.38 ppm), the methylene CH_2-3 (δ_{13C} 21.8; δ_{1H} 2.76/2.26 ppm), and the deshielded methylene CH_2-4 (δ_{13C} 54.9; δ_{1H} 4.21/3.51 ppm), were sequentially connected through COSY correlations, defining an independent spin system. The isolated olefinic methine CH-6 (δ_{13C} 100.9; δ_{1H} 6.21 ppm) showed HMBC correlations to CH-2, as well as to the deshielded enamine-like non-protonated sp^2 carbon C-5 (δ_{13C} 177.3) and the ketone carbonyl C-1 (209.0). Together, these correlations established a continuous fragment comprising C-5, CH-6, C-1 and CH-2, which extended through CH_2-3 and CH_2-4 The ^1H-^{13}C HMBC correlation between H_2-4 and C-5 was important to support the presence of a cyclic substructure. Closure of the remaining degree of unsaturation of CH-2 through the nitrogen atom, together with the ^1H-^{15}N HMBC correlations observed from H-6 and H_2-4 to the same nitrogen nucleus, as well as the deshielded chemical shifts of CH_2-4 and CH-2, allowed this second fragment to be assigned as the bicyclic system 2-amino-1-azabicyclo[3.2.0]hept-2-en-4-one. Although the ^1H-^{15}N HMBC did not allow us to establish the connection between this heterocycle and the

unsaturated side tail, this linkage was inferred from MS/MS fragmentation analysis, fragments **a** and **b** (**Table S4**).

<u>Metaze B</u> was detected as a unique feature at *m/z* 263.1026 $[M+H]^+$, consistent with the molecular formula $C_{13}H_{14}N_2O_4$ (**Figure S9**). Detailed analysis of ^1H, ^{13}C and HSQC experiments revealed the presence of four sp^2 methines, one sp^2 methylene, one deshielded sp^3 methine, one deshielded sp^3 methylene, and one sp^3 methyl group. Combined interpretation of ^{13}C and ^1H-^{13}C HMBC spectra enabled the identification of three non-protonated sp^2 carbons, two of them deshielded, as well as two carbonyl carbons bonded to heteroatoms. Using the exomethylene signals as the starting point for structural elucidation, the same short (*E*)-4-methylpenta-2,4-dienamide side chain observed in Metaze A was identified, extending from C-8 to CH_3-13 (**Table S5**). The remainder of the molecule is elucidated through two distinct ^1H-^1H COSY spin systems: the sp^2 methines CH-3 (δ_{13C} 100.6; δ_{1H} 6.33 ppm) and CH-4 (δ_{13C} 103.2; δ_{1H} 5.52 ppm) likely in a *Z* configuration ($^3J_{H3,H4}$ 6.3 Hz); and the deshielded methine CH-6 (δ_{13C} 57.9; δ_{1H} 5.05 ppm) coupled to the deshielded methylene CH_2-7 (δ_{13C} 60.3; δ_{1H} 4.27/3.84 ppm). ^1H-^{13}C HMBC correlations from H-3/H-4 to the deshielded carbons C-2 (δ_{13C} 139.2 ppm) and C-5 (δ_{13C} 149.4) established that this pair of olefinic protons was located between two non-protonated sp^2 carbons bonded to heteroatoms. The ^1H-^{13}C HMBC correlation from H-4 to C-6 further extended the fragment from C-5 to CH-6 and CH_2-7. In addition, ^1H-^{15}N HMBC correlations from H-4, H-6 and H-7 to the same nitrogen nucleous established the presence of a fused azetidine-containing bicyclic system analogous to that observed in Metaze A. The long-range heteronuclear correlation between H_2-7 and the carbonyl C-1 (δ_{13C} 157.9) whose chemical shift is consistent with a carbamate carbonyl, ultimately supported assignment of the heterobicyclic system 4-amino-8-hydroxy-3-oxa-1-azabicyclo[5.2.0]nona-4,6-dien-2-one as the second structural moiety of Metaze B. The connectivity between the short side chain and this bicyclic system was established by MS/MS fragmentation analysis. The involvement of nitrogen substitution at C-2 and C-5 was also supported by their characteristic ^{13}C chemical shifts (**Table S5**).

Table S1. Soil sample and metagenomic library information.

Soil sample	Subpools	Clones per subpool	Library clones	Avg. Insert size (kbp)	Total library size (Gbp)	Reads number			OTU		
						AD	KS	ClpP	AD	KS	ClpP
OR13	768	25,000	2E+07	40	800	364,169	233,111	10,587,590	134,496	160,021	28,706

Table S2. Primers used in this study.

Primer	Sequence (5'-3')	Purpose
Deg-ClpP-F2	TACATYMAYWSSCCSGGYGG	Soil Library screening for *clpP* gene
Deg-ClpP-R2	AYSYSGTCGAYSADBCCGWA	Soil Library screening for *clpP* gene
AD3-F	SATBTAYACSTCVGGHWCSAC	Soil library screening for AD-domain
AD3-R	CCANRTCNCCBGTSYKGTASA	Soil library screening for AD-domain
KS3-F	TGYTCSDSSTCGCTSGTSGCS	Soil library screening for KS-domain
KS3-R	GTNCCSGTSCCRTGBGCYTCS	Soil library screening for KS-domain
OR13-ClpP-F	TCTACGACACGATGAACCACAT	Cosmid recovery
OR13-ClpP-R	TCAGATGTTGGTGATGATCCGG	Cosmid recovery
OR13-AD-F	GTGTGGGAGCTGTTCTGGTG	Cosmid recovery
OR13-AD-R	GCAGCTTGATGTTGTCGATGG	Cosmid recovery
MTA-TCV-FL	ACCCTGCAGGAGCTCGCATGGAGGATCCCCTGACCGAGC	TAR capture vector construction for the MTA BGC
MTA-TCV-RL	AACTTCGGTTTAAACTGACGGTGTAGAGCAGCAG	TAR capture vector construction for the MTA BGC
MTA-TCV-FR	CGTCAGTTTAAACCGAAGTTCGACCTGGTGTTCAG	TAR capture vector construction for the MTA BGC
MTA-TCV-RR	GAGGCTAGCCCTAACGCATGGTCCTCGATCATGTAGGCGATC	TAR capture vector construction for the MTA BGC
psf-ClpP1-F	GTTGCATCAGACAGGCGGTGCGGGTACCGTGAGCCAAGTGACTGACATGCGTTCG	Mtb ClpP1 overexpression
psf-ClpP1-R	CCAAGCTCAGCTAATTAAGCTTACTACTGTGCTTCTCCATTGACGTGGGCGCGGGTG	Mtb ClpP1 overexpression
psf-ClpP2-F	GTTGCATCAGACAGGCGGTGCGGGTACCGTGAATTCCCAAAATTCTCAGATCCAGCCC	Mtb ClpP2 overexpression
psf-ClpP2-R	CCAAGCTCAGCTAATTAAGCTTACTAGGCGGTTTGCGCGGAGAGCTTCCGGTA	Mtb ClpP2 overexpression
OR13_SNP11_UP	GGTGCTGCCCACGACCTTCGTCGCCCGCCGTTCGGGTTGATCACAACCCTCCTAGTAACG	Refactoring for the MTA BGC
OR13_SNP11_DN	TGAGCGGGACAGGACGAGATAACTCCGAGTGCGGTGCCATTAATTCACCTCCTGAGGCTT	Refactoring for the MTA BGC

Table S3. Strains and constructs used in this study.

Strains	Description	Source/Reference
E. coli EPI300	General cloning and plasmid maintenance	Biosearch Technologies
E. coli S17-1	Conjugation donor	ATCC
E. coli BL21 (DE3)	ClpP1, ClpP2, and ClpC1 overexpression host	Gold Biotechnology
S. albus J1074	Heterologous expression host for BGCs	ATCC
S. cerevisiae BY4727	Cloning and BAC maintenance	[6]
**Constructs**		
pWEB::TNC	Metagenomic library backbone	Epicentre Biotechnologies
pET-28a(+)	Mtb ClpC1 overexpression backbone	Novagen
pSF1389	Mtb ClpP1 and ClpP2 overexpression backbone	Addgene
pTAR-lys	The CPA BGC assembly backbone	[7]
pTAR-TCV-MTA	Capture vecotor for the MTA BGC assembly	This study
pTAR-MTA	MTA BGC captured on pTAR-lys backbone	This study
pTAR-MTA-SNP11	Engineered MTA BGC captured on pTAR-lys backbone	This study

Table S4. NMR data for metaze A in CD_3OD (^1H 600 MHz and ^{13}C 150 MHz at 298 K). Structure of Metaze A: bold bonds equal ^1H-^1H COSY correlations, blue arrows ^1H-^{13}C and red arrows ^1H-^{15}N HMBC correlations.

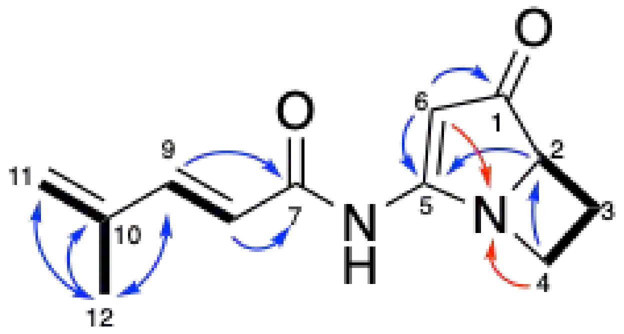

#	Type	^{13}C	^1H	m, *J*(Hz)
1	C	209.0		
2	CH	68.6	4.38	dd, 9.9; 6.2
3	CH_2	21.8	2.76	m
			2.26	m
4	CH_2	54.9	4.21	q, 17.8; 8.9
			3.51	td, 5.4; 9.8; 9.8
5	C	177.3		
6	CH	100.9	6.21	s
7	C	166.1		s
8	CH	120.9	6.20	d, 15.5
9	CH	148.2	7.47	d, 15.5
10	C	141.8		
11	CH_2	126.1	5.47	s
			5.46	s
12	CH_3	18.2	1.94	s

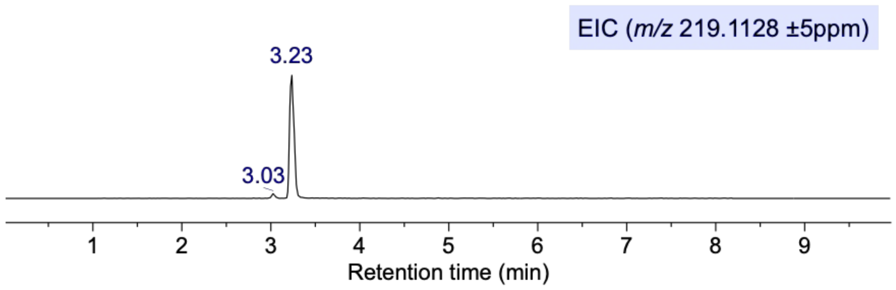

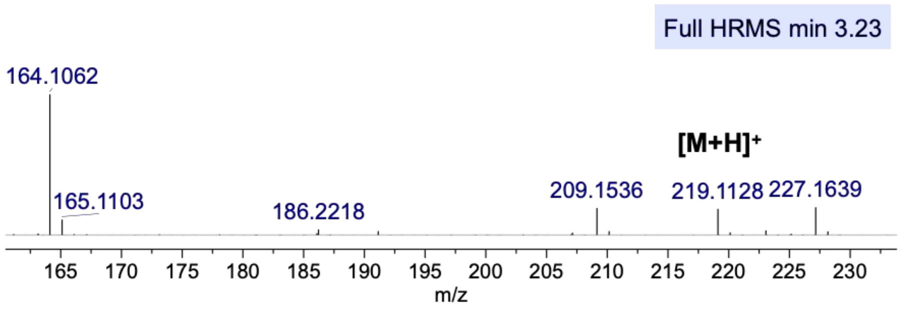

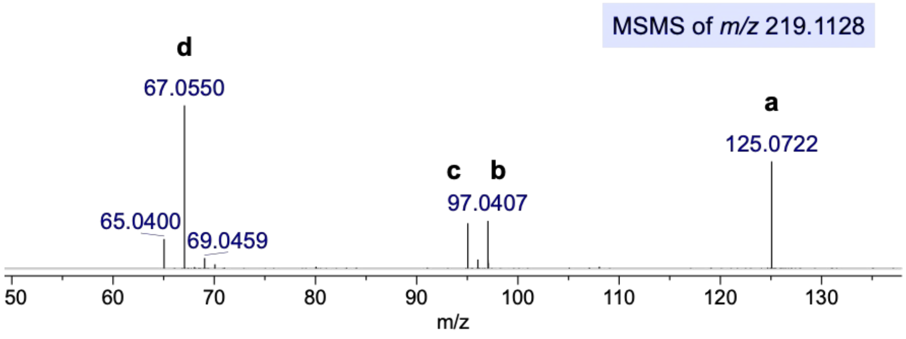

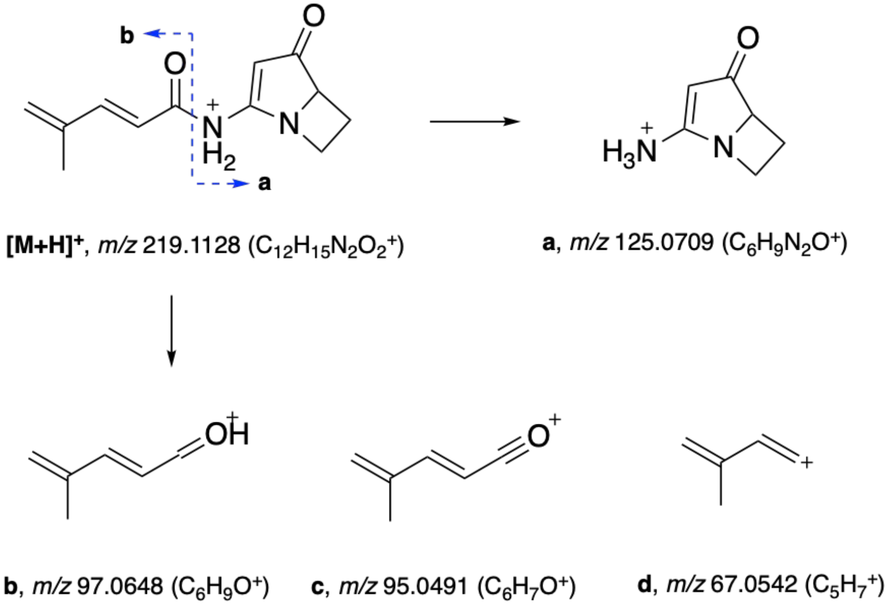

Precursor ion annotation:

Ion	Formula	*m/z* obs.	*m/z* theo.	RDB	Δppm
[M+H]+	$C_{12}H_{15}N_2O_2^+$	219.1128	219.1128	7.0	0.12

MS/MS fragment annotation

Clv.	Formula	*m/z* obs.	*m/z* theo.	RDB	Δppm
a	$C_6H_9N_2O^+$	125.0722	125.0709	4.0	10.39
b	$C_6H_9O^+$	97.0407	97.0648	3.0	>20
c	$C_6H_7O^+$	95.0499	95.0491	3.5	-8.41
d	$C_5H_7^+$	67.0550	67.0420	2.5	>20

Figure S1. Full HRMS and MS/MS analysis for metaze A.

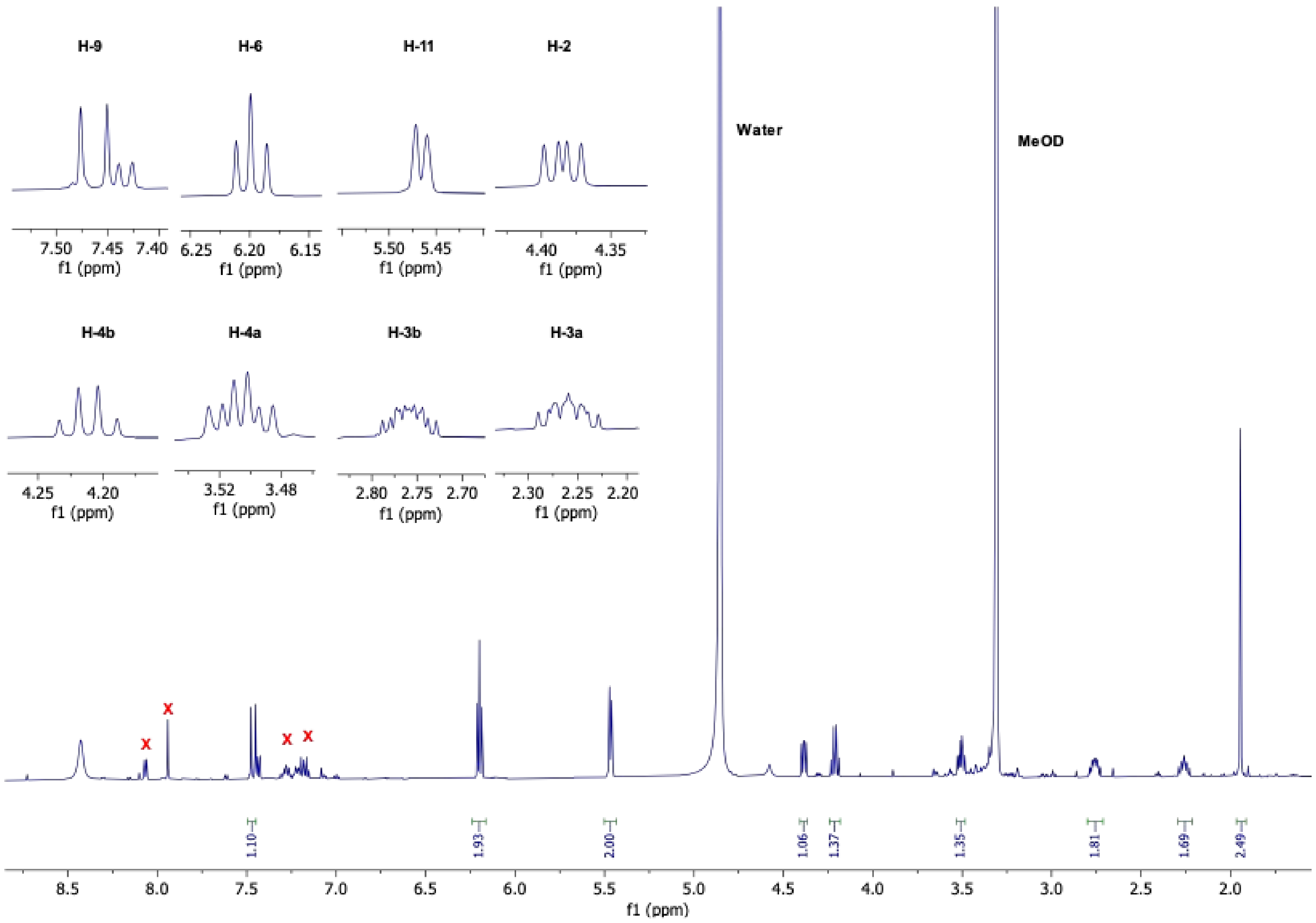

Figure S2. ^{1}H NMR spectrum for metaze A in CD_3OD (600 MHz, 298K). Red “X” marks contaminant peaks.

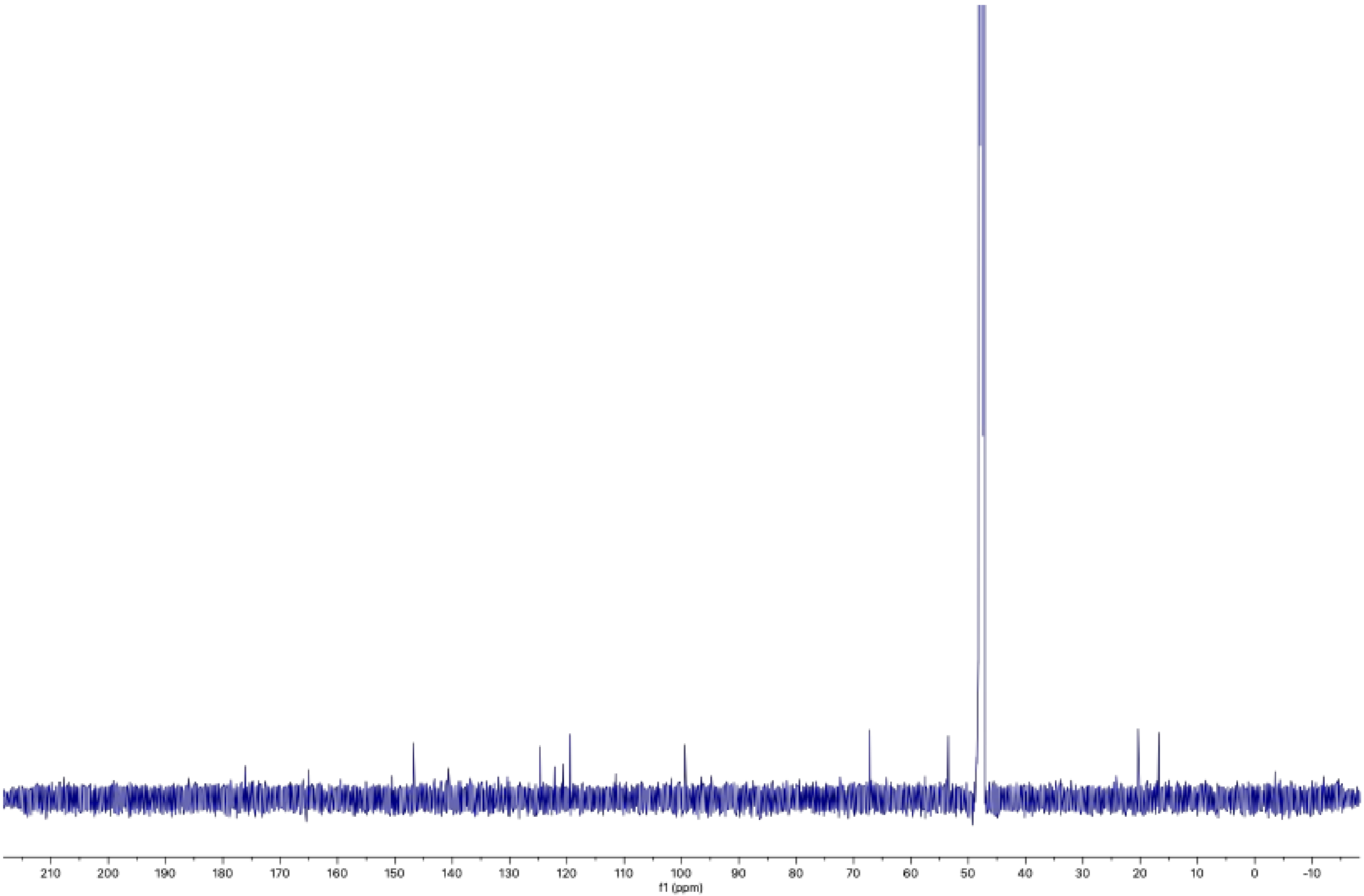

Figure S3. ^{13}C NMR spectrum for metaze A in CD_3OD (150 MHz, 298K).

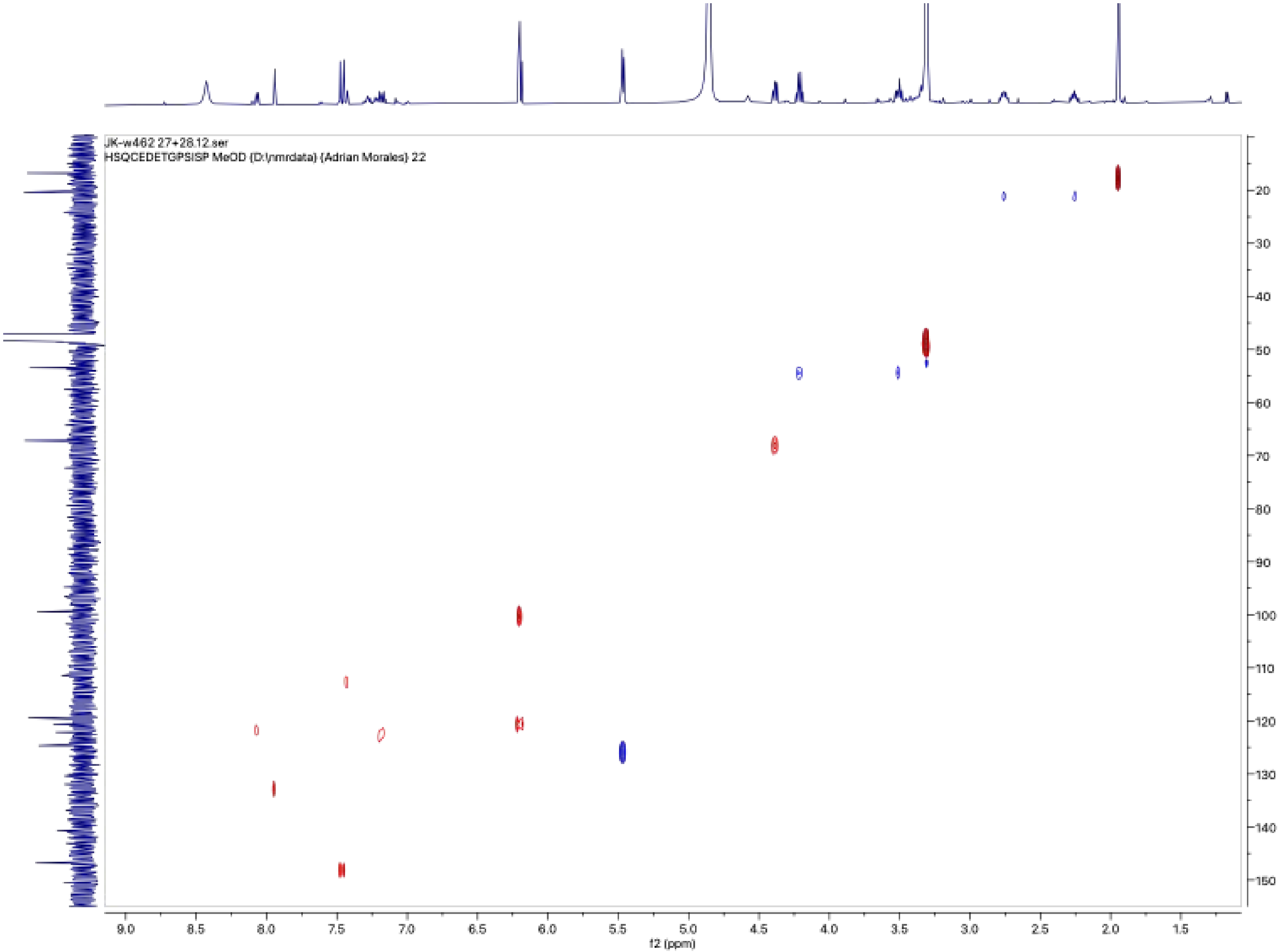

Figure S4. HSQC$_{ed}$ spectrum for metaze A in CD_3OD (600 MHz, 298K).

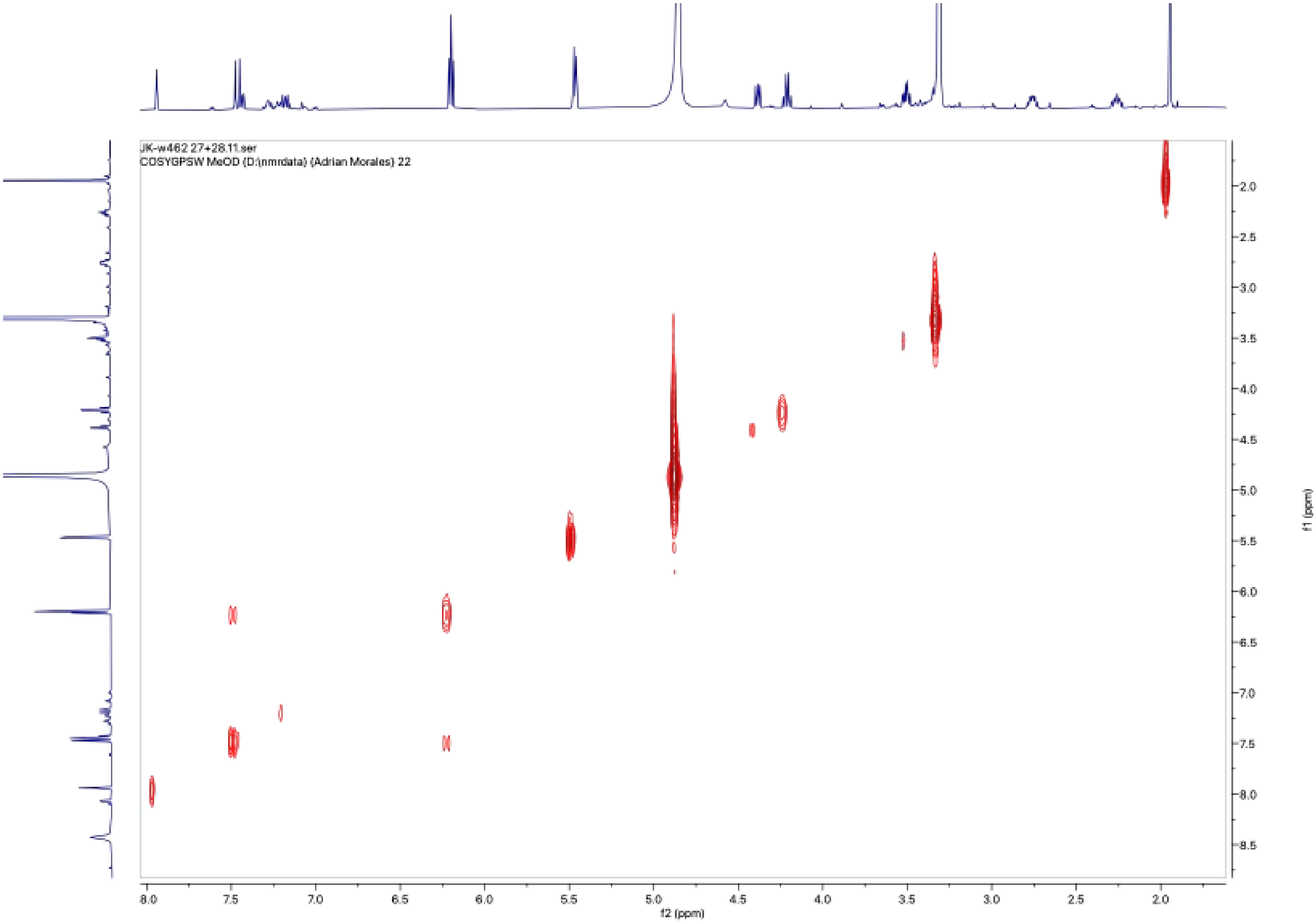

Figure S5. COSY spectrum for metaze A in CD_3OD (600 MHz, 298K).

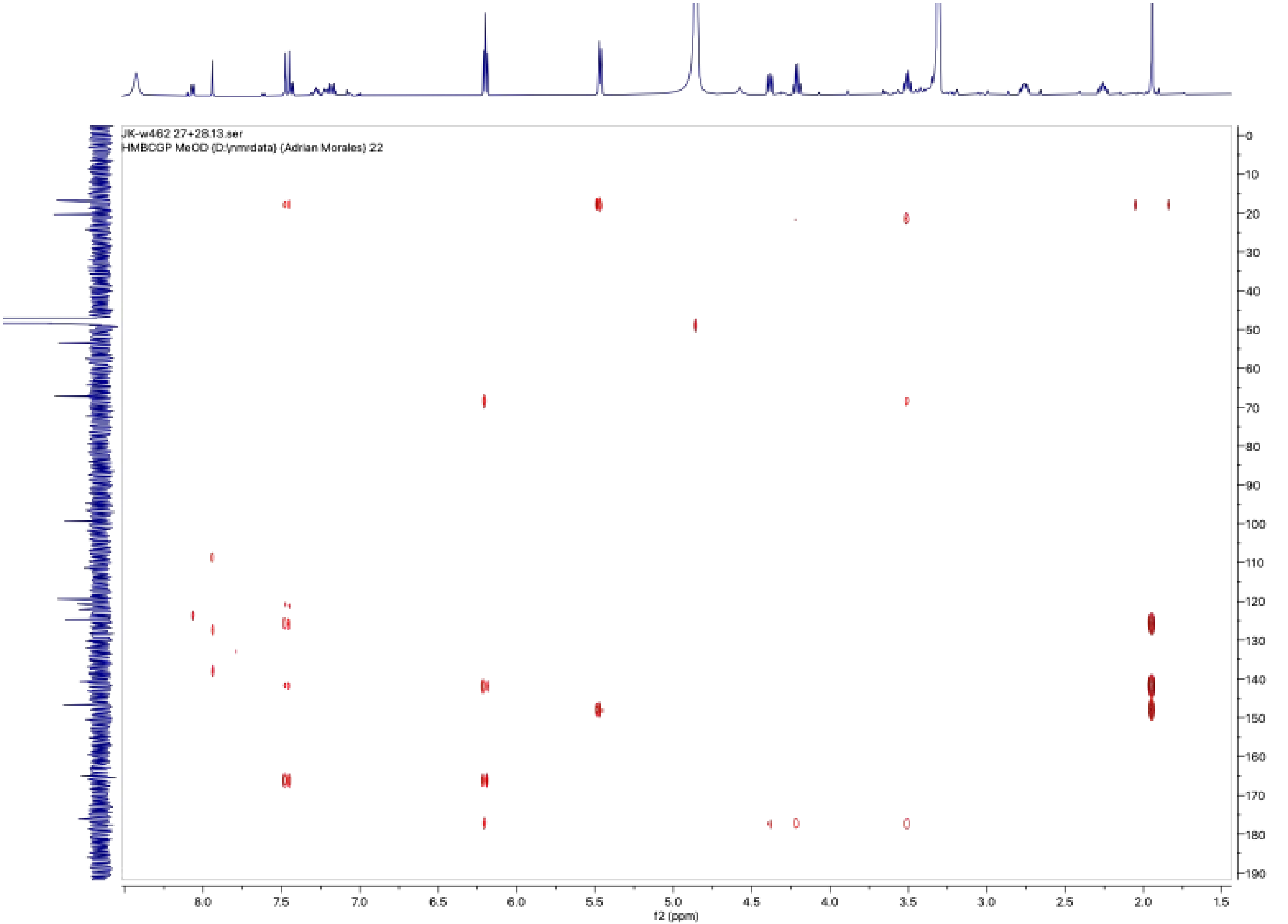

Figure S6. ^{1}H-^{13}C HMBC spectrum for metaze A in CD_3OD (600 MHz, 298K).

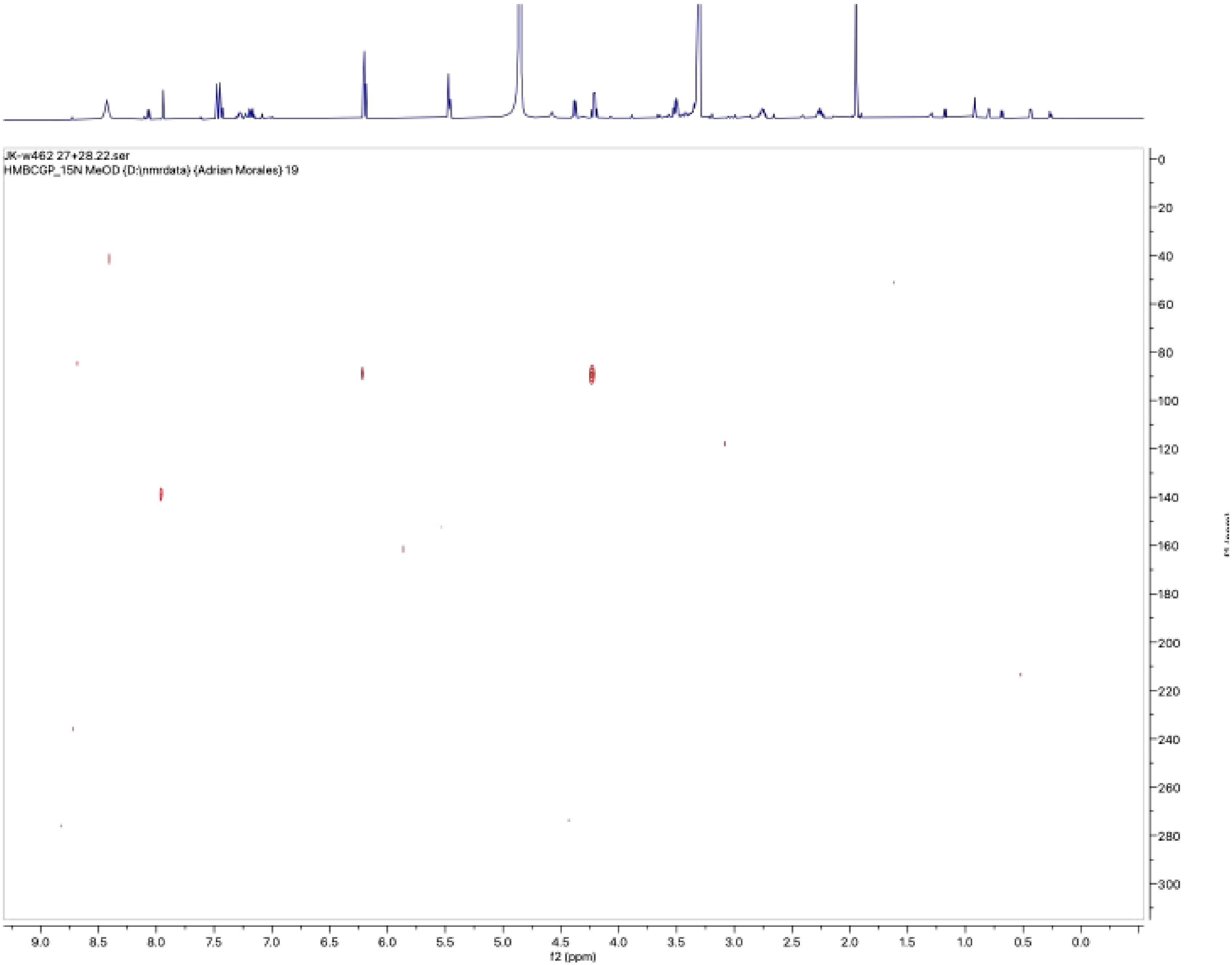

Figure S7. ^{15}N-^{13}C HMBC spectrum for metaze A in CD_3OD (600 MHz, 298K).

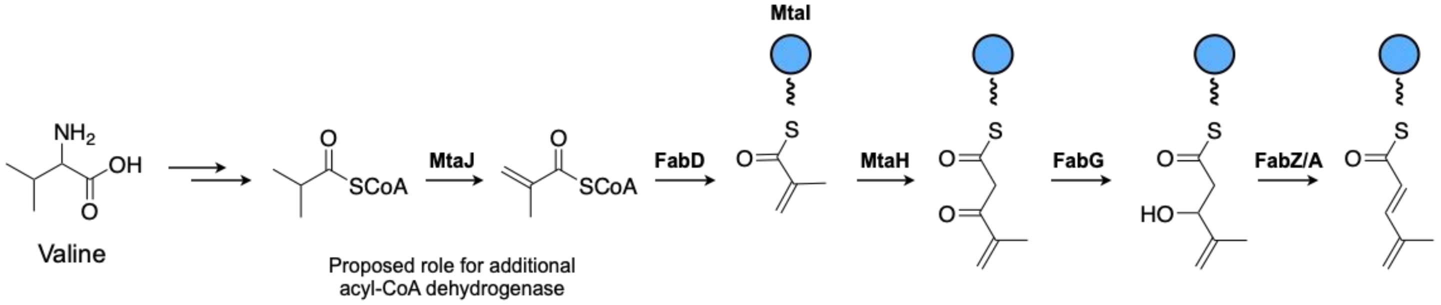

Figure S8. Proposed biosynthesis of the acyl tail of metaze A/B.

Table S5. NMR data for metaze B in CD_3OD (^1H 600 MHz and ^{13}C 150 MHz at 298 K). Structure of Metaze B: bold bonds equal ^1H-^1H COSY correlations, blue arrows ^1H-^{13}C and red arrows ^1H-^{15}N HMBC correlations.

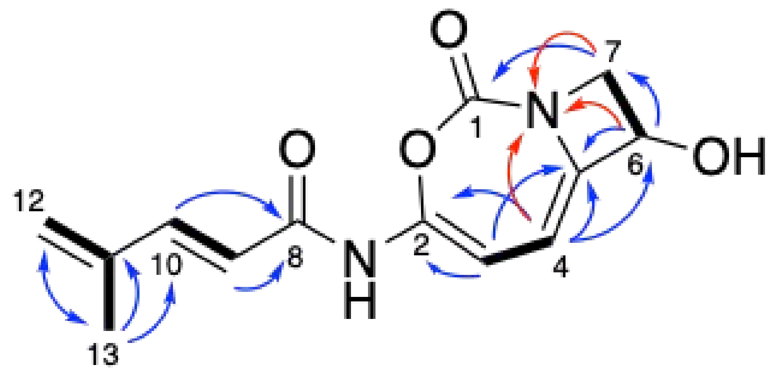

#	Type	^{13}C	^1H	m, *J*(Hz)
1	C	157.9		
2	C	139.2		
3	CH	100.6	6.33	d, 6.3
4	CH_2	103.2	5.52	*d*, 6.3
5	C	149.4		
6	CH	57.9	5.05	br(t)
7	CH_2	60.3	3.84	*d*, 3.6; 8.6
			4.27	br(t), 7.5
8	C	166.4		
9	CH	121.8	6.11	d, 15.5
10	CH	145.9	7.31	d, 15.5
11	C	142.2		
12	CH_2	124.5	5.37	br(d)
13	CH_3	18.3	1.91	s

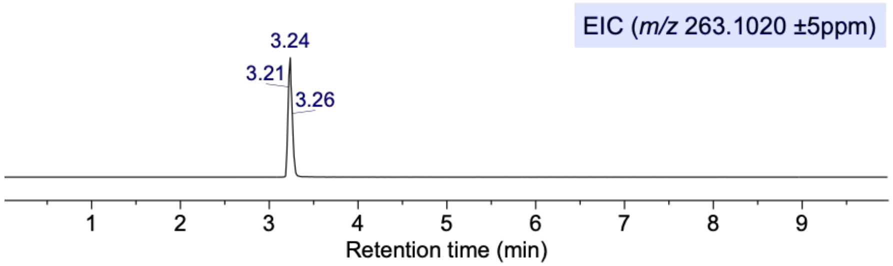

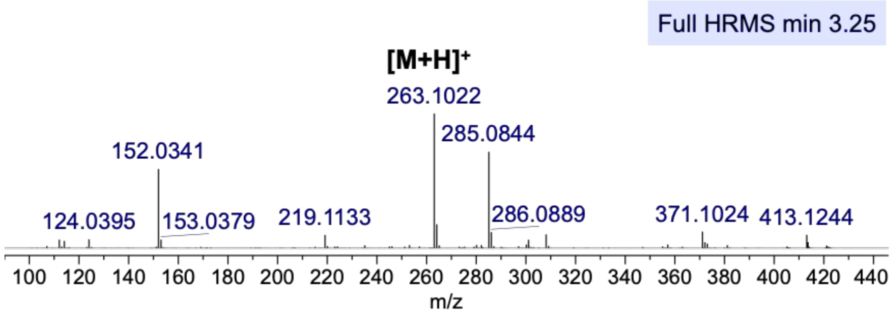

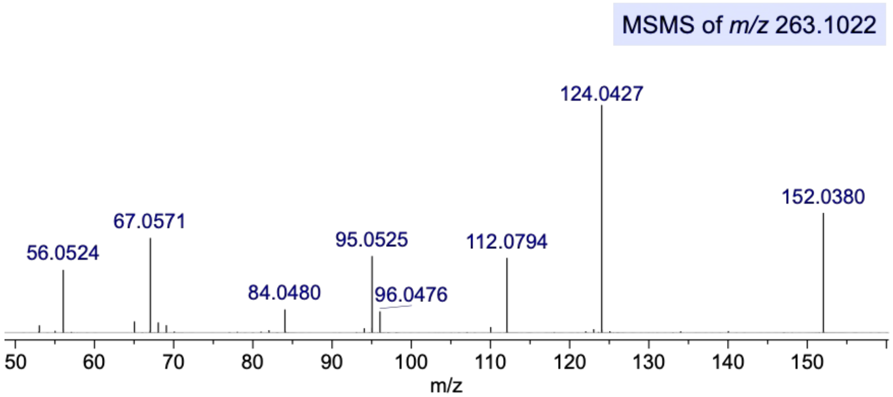

Figure S9. Full HRMS and MSMS analysis for metaze B.

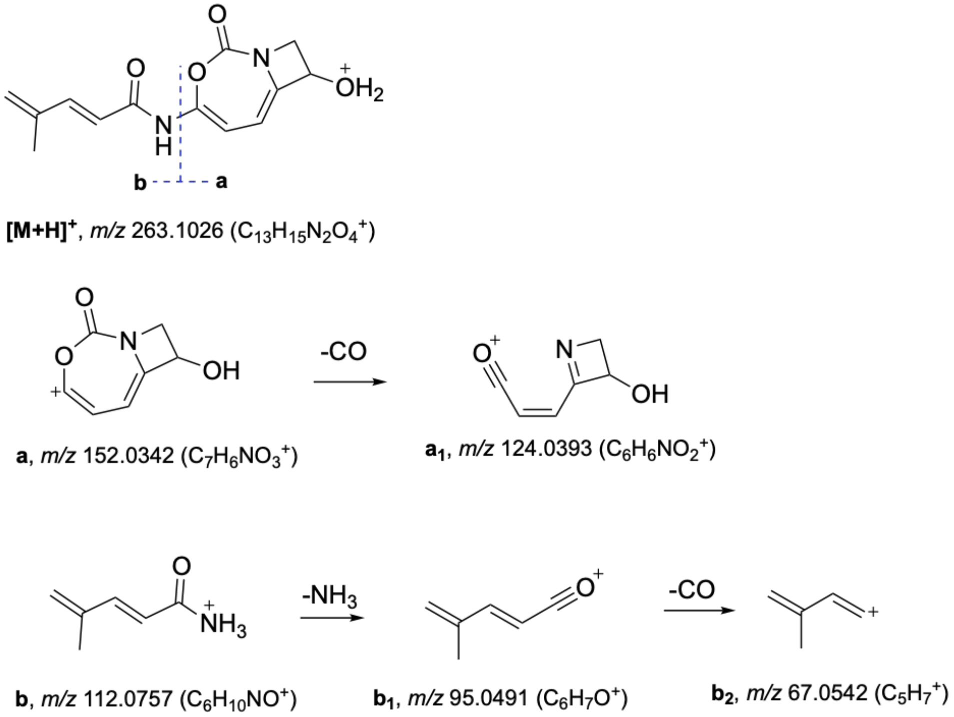

Precursor ion annotation:

Ion	Formula	*m/z* obs.	*m/z* theo.	RDB	Δppm
[M+H]+	$C_{13}H_{15}N_2O_4^+$	263.1022	263.1026	8.0	-1.50

MS/MS fragment annotation

Clv.	Formula	*m/z* obs.	*m/z* theo.	RDB	Δppm
a	$C_7H_6NO_3^+$	152.0380	152.0342	5.5	>20
a_1	$C_6H_6NO_2^+$	124.0427	124.0393	4.5	>20
b	$C_6H_{10}NO^+$	112.0794	112.0757	2.5	>20
b_1	$C_6H_7O^+$	95.0525	95.0491	3.5	>20
b_2	$C_5H_7^+$	67.0571	67.0542	2.5	>20

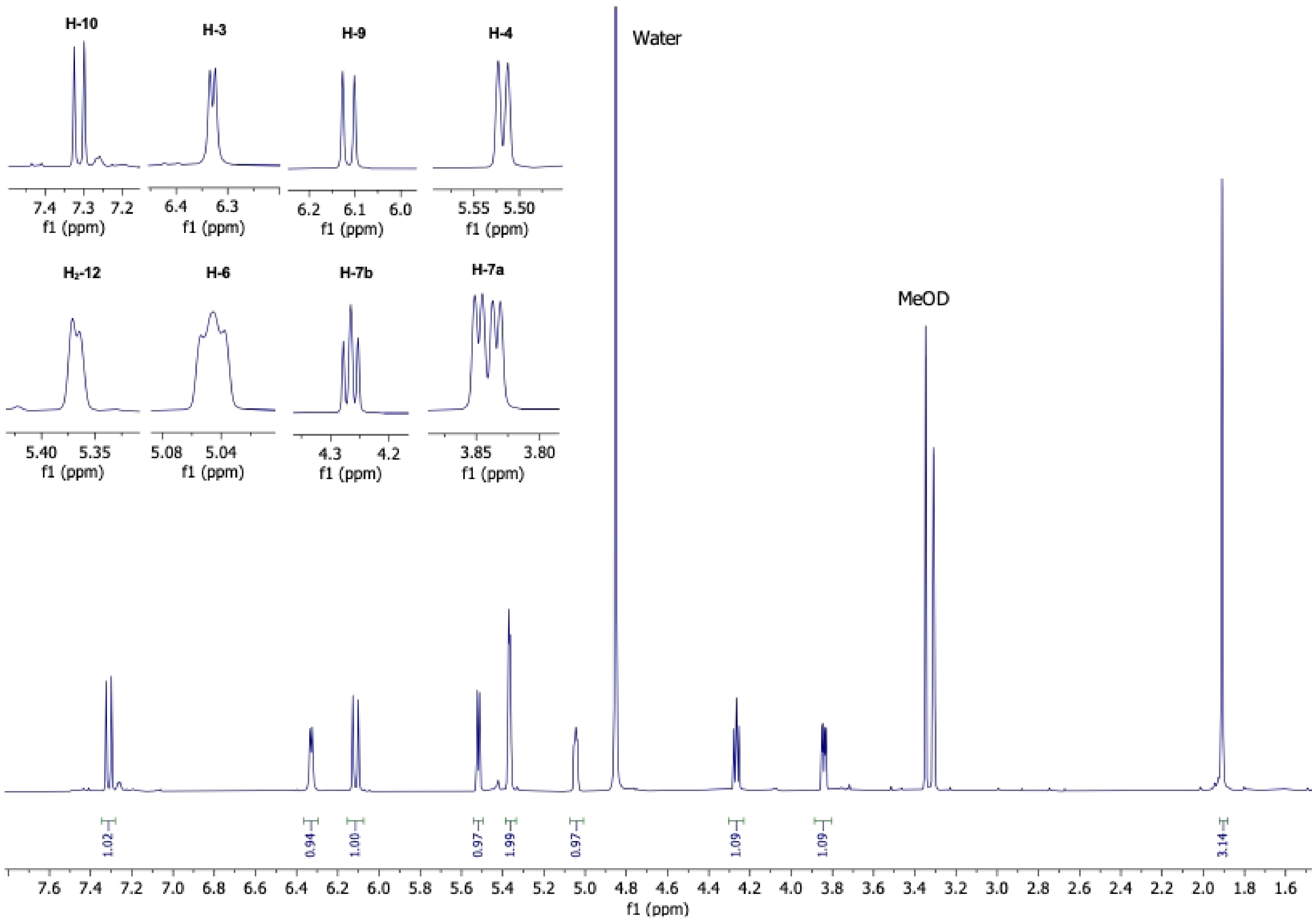

Figure S10. ^{1}H NMR spectrum for metaze B in CD_3OD (600 MHz, 298K).

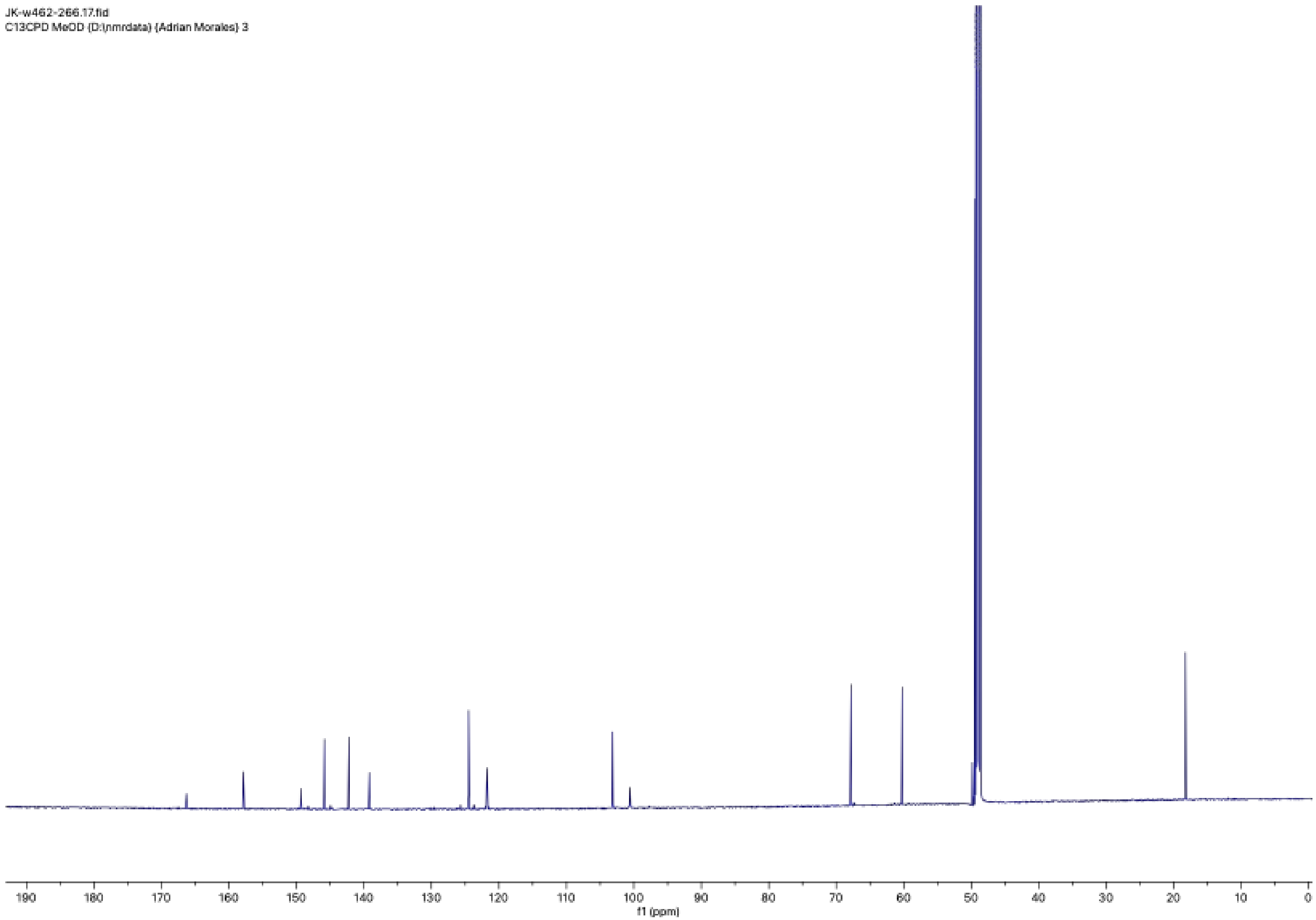

Figure S11. ^{13}C NMR spectrum for metaze B in CD_3OD (150 MHz, 298K).

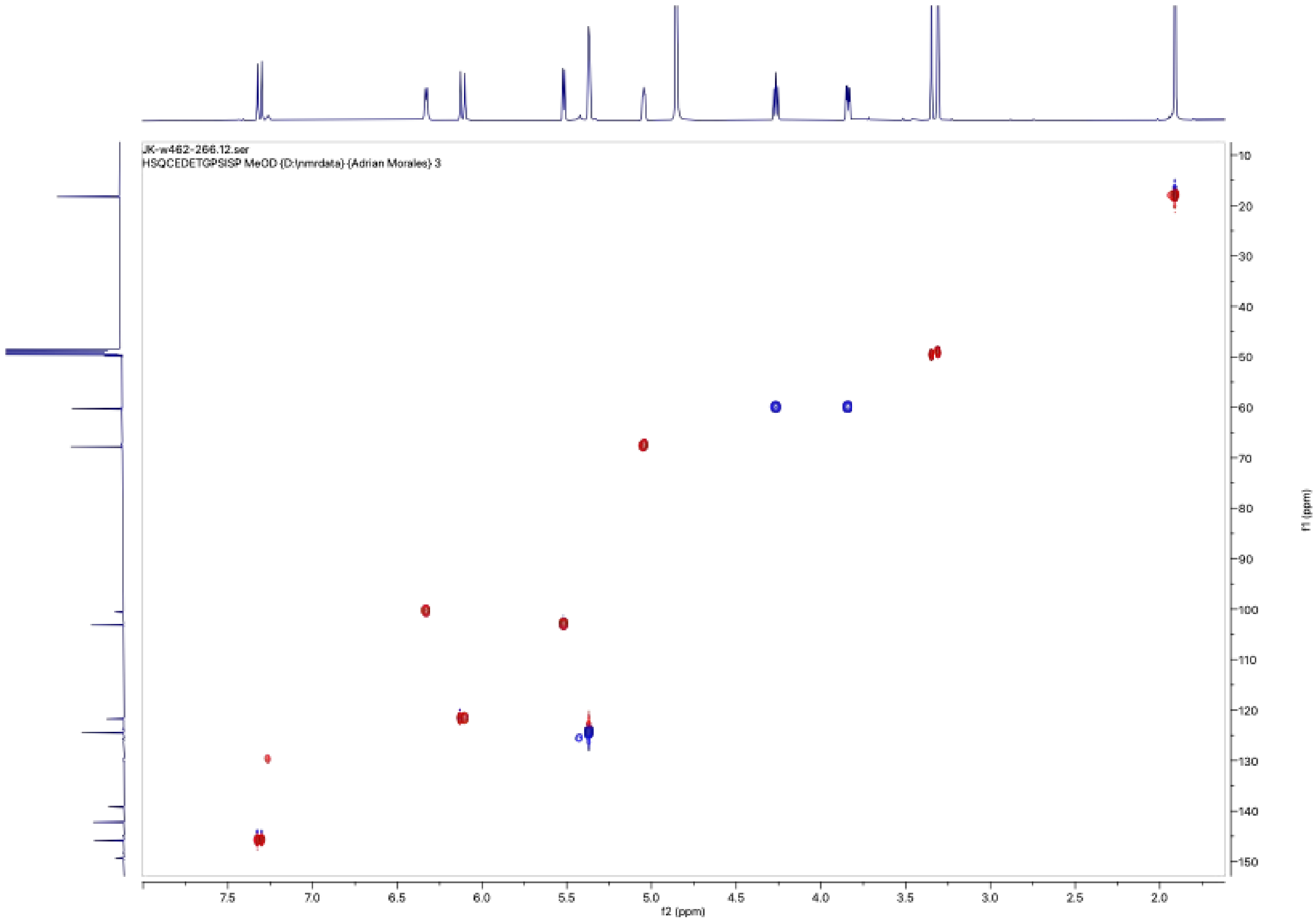

Figure S12. HSQC$_{ed}$ spectrum for metaze B in CD_3OD (600 MHz, 298K).

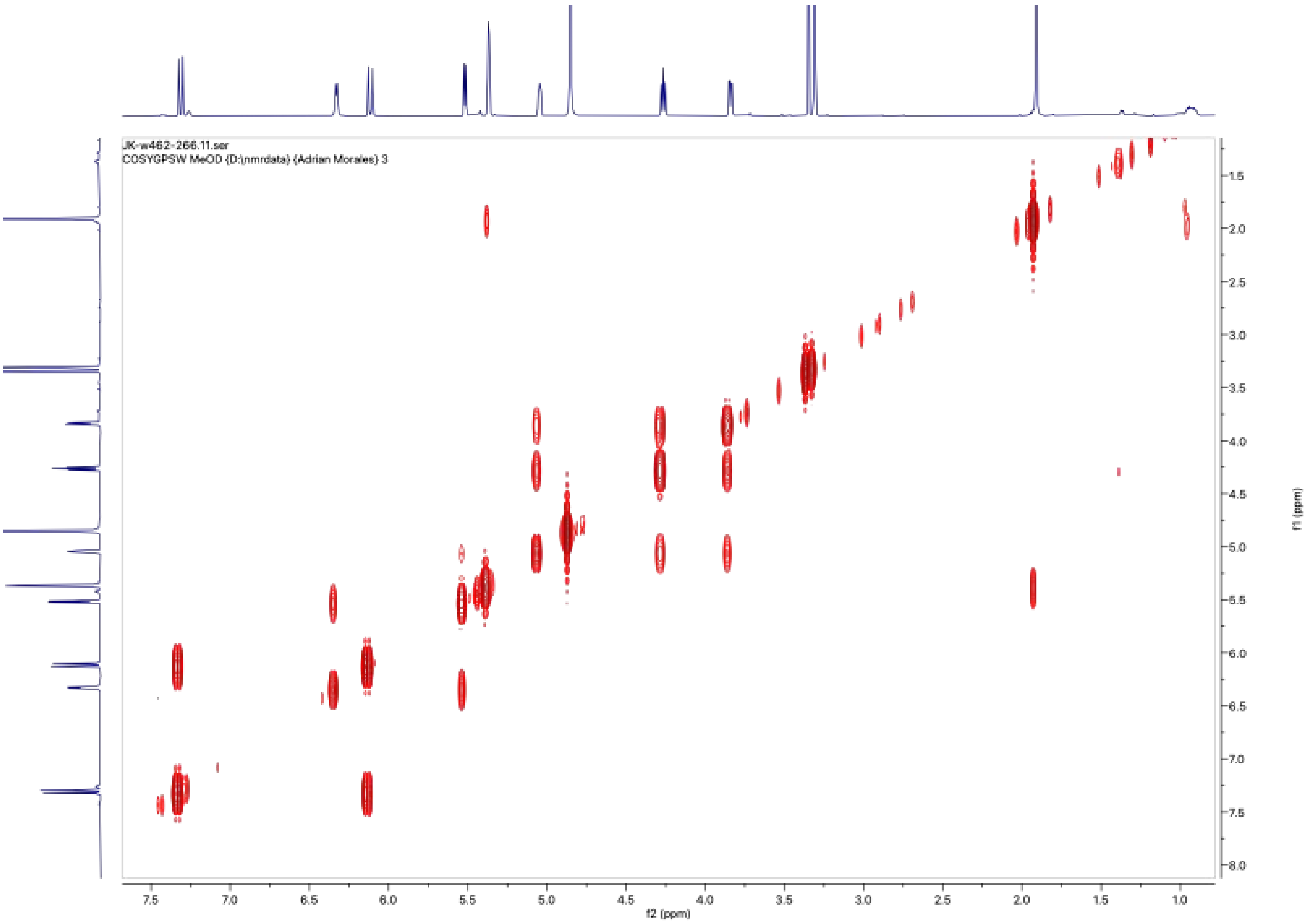

Figure S13. COSY spectrum for metaze B in CD_3OD (600 MHz, 298K).

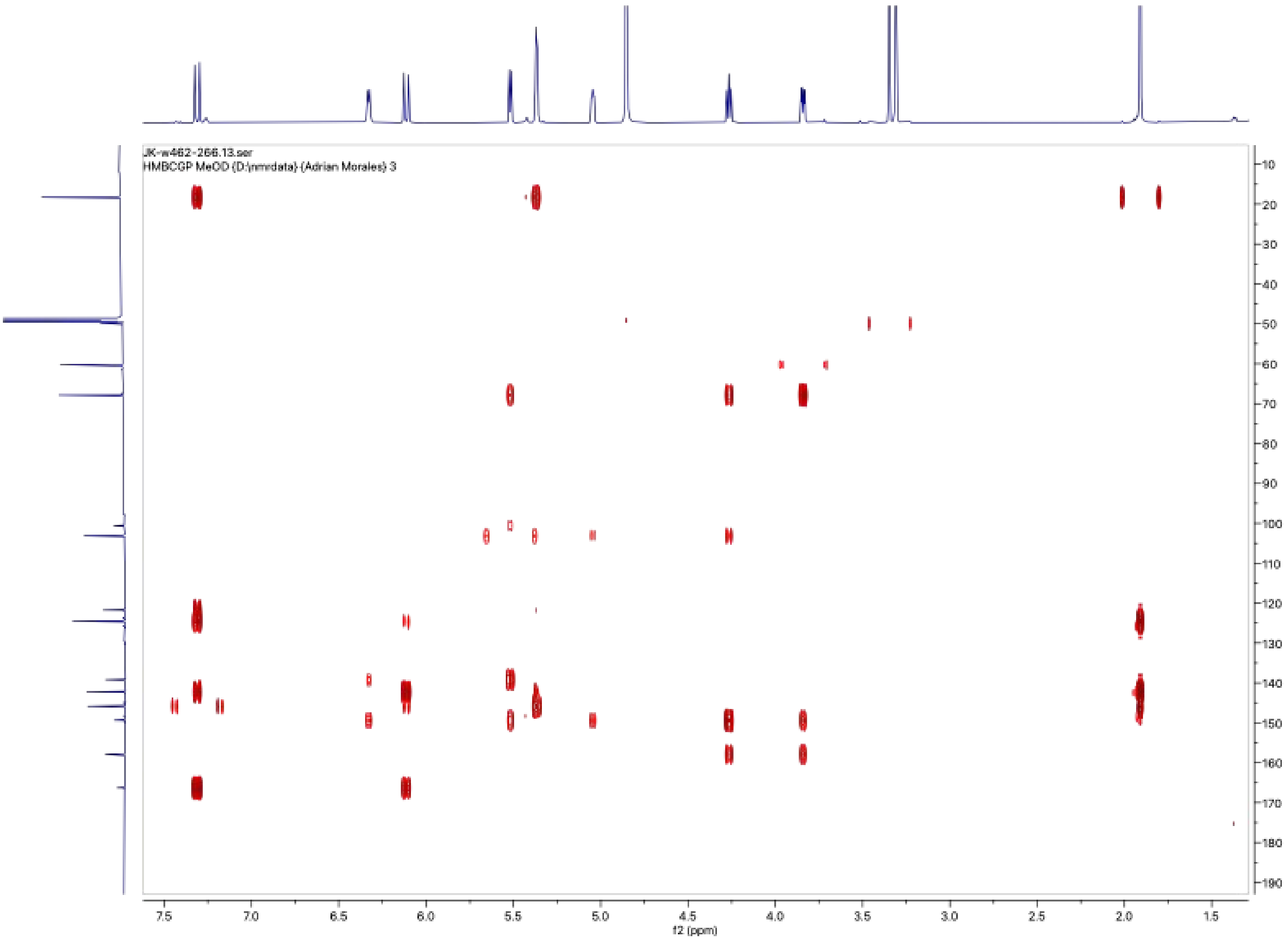

Figure S14. ^{1}H-^{13}C HMBC spectrum for metaze B in CD_3OD (600 MHz, 298K).

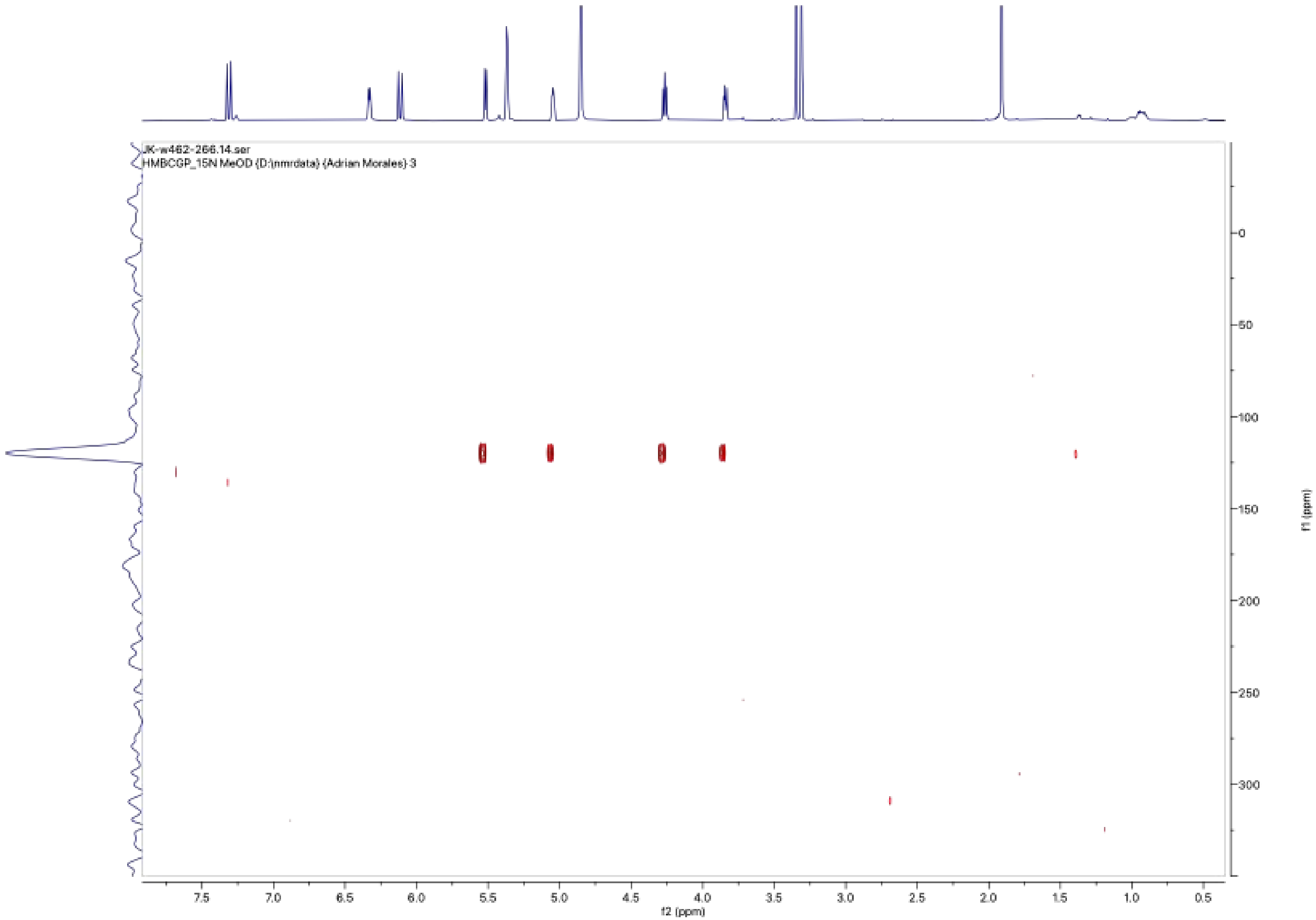

Figure S15. ^{15}N-^{13}C HMBC spectrum for metaze B in CD_3OD (600 MHz, 298K).

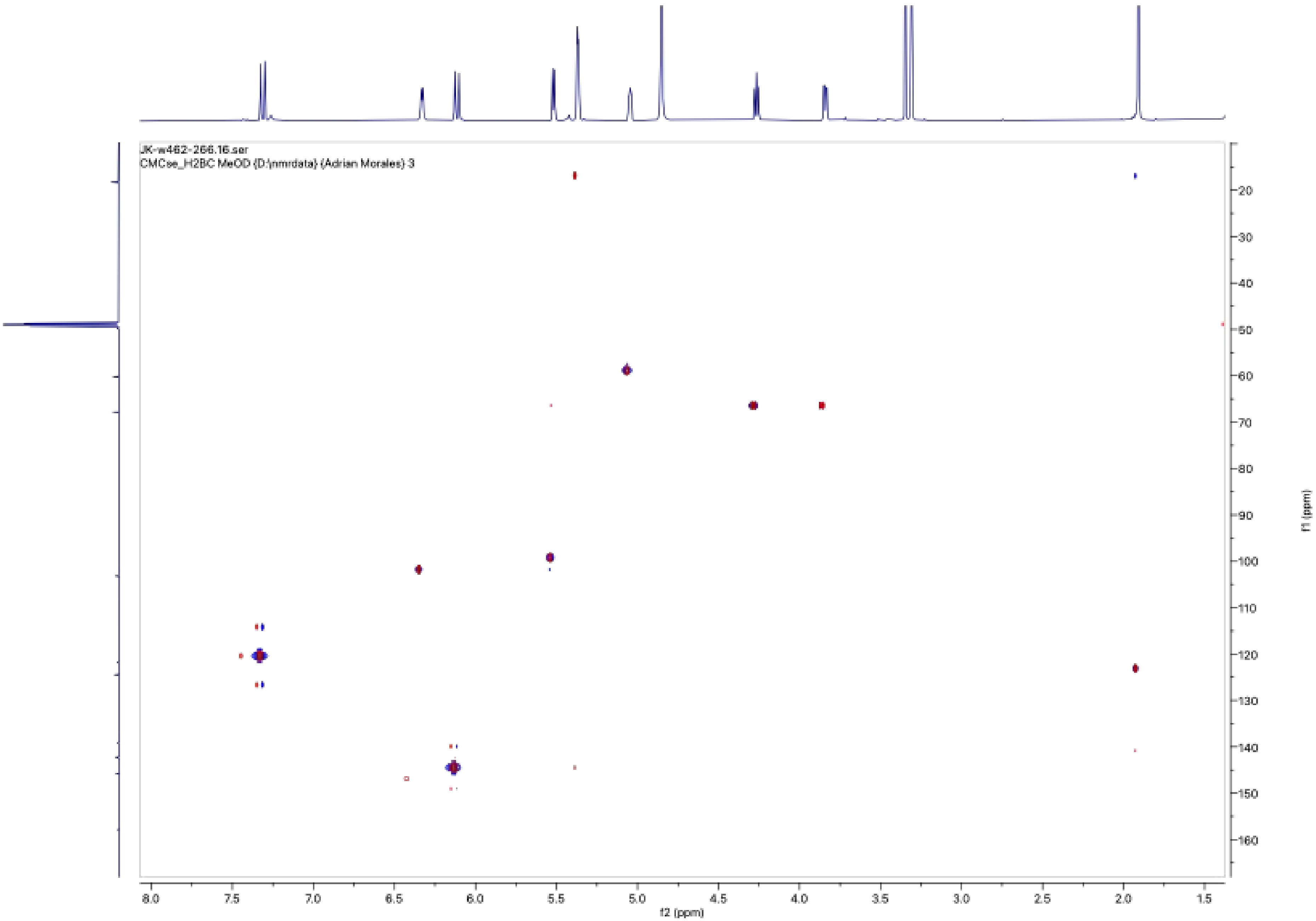

Figure S16. H2BC spectrum for metaze B in CD_3OD (600 MHz, 298K).

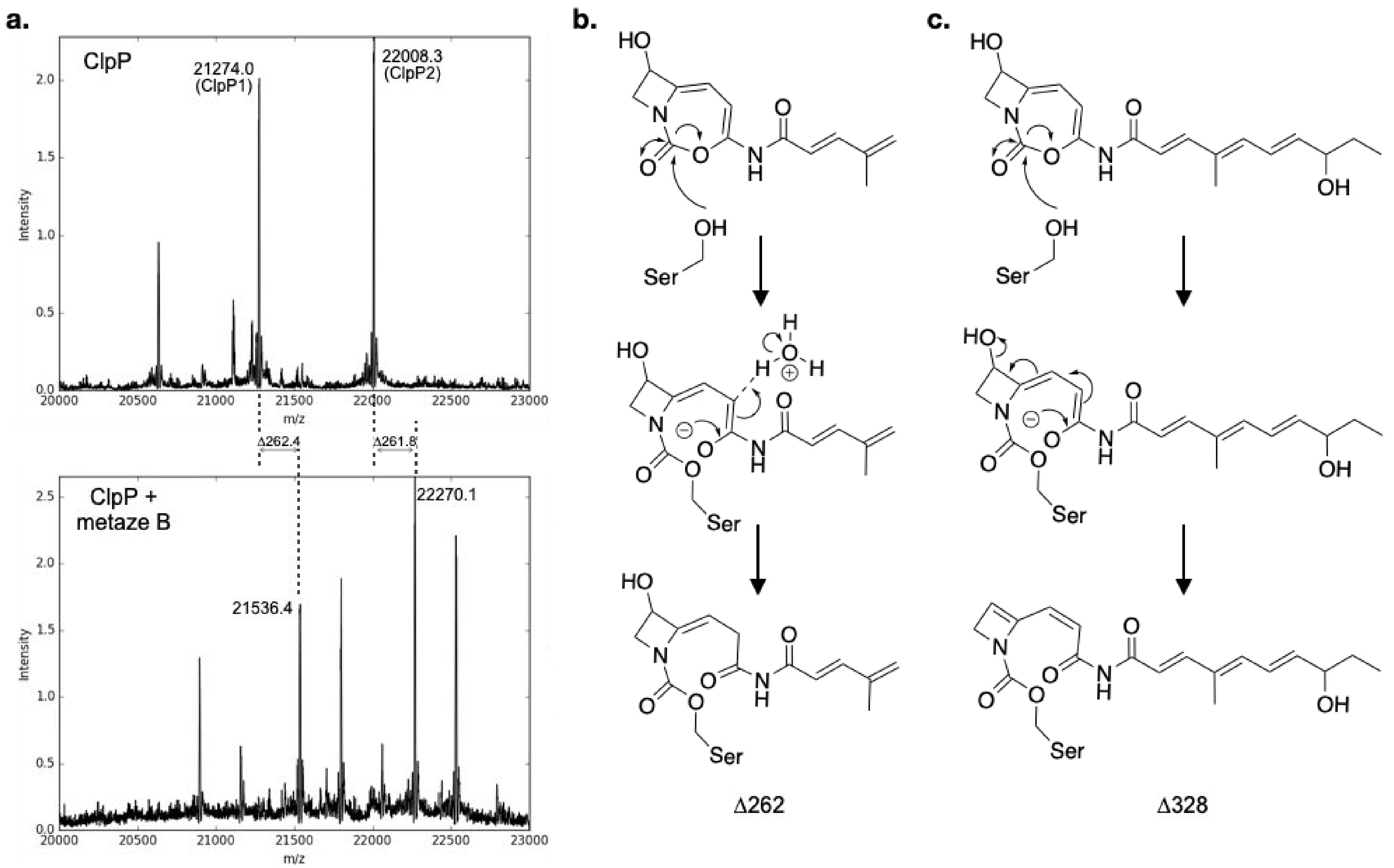

Figure S17. Metaze B covalently modifies and inhibits Mtb ClpP. a. Intact protein LC-MS. **b.** Proposed reaction of metaze B with Mtb ClpP's active site Ser. **c.** Proposed reaction of clipibicyclene with *Streptomyces cattleya* ClpP's active site Ser. The difference in proposed reaction products may reflect different positioning of the inhibitors within distinct ClpP catalytic sites. Clipibicyclene was assayed against *Streptomyces cattleya* and *E. coli* ClpP[8], whereas metaze B was assayed against Mtb ClpP. Elucidation of the mechanistic details will require additional structural studies.

Table S6. Inhibition activities of metaze A and B.

	MIC (μg/ml)	
**Organism**	Metaze A	Metaze B
**Gram positive**		
Staphylococcus aureus SH1000	>32	>32
Staphylococcus epidermidis W23144	>32	>32
Streptococcus pyogenes ATCC 19615	>32	>32
Streptococcus pneumoniae TCH8431	>32	>32
Enterococcus faecium Com15	>32	>32
Enterococcus faecalis EF16	>32	>32
Mycobacterium tuberculosis mc^2 6206	>32	>32
Mycobacterium smegmatis mc^2 155	>32	>32
Micrococcus luteus NRRL B-1018	>32	>32
**Gram negative**		
Pseudomonas aeruginosa PAO1	>32	>32
Enterobacter cloacae ATCC 13047	>32	>32
Klebsiella pneumoniae ATCC 10031	>32	>32
Acinetobacter baumannii ATCC 17978	>32	>32
**Fungus**		
Candida albicans ATCC 18804	>32	>32
**Human**		
Homo sapiens HEK293	>32 (IC_{50})	>32 (IC_{50})

References:

(1) Kan, J.; Spotton, K.; Morales-Amador, A.; Hernandez, Y.; Burian, J.; Panfil, C.; Ternei, M. A.; Boer, R. E.; Bhattacharjee, A.; Brady, S. F. Mode of action guided metagenomic natural product discovery reveals convergent evolution of a ClpP-targeting motif. *Nature Communications* **2026**, *In press*.
(2) Kim, J. H.; Feng, Z.; Bauer, J. D.; Kallifidas, D.; Calle, P. Y.; Brady, S. F. Cloning large natural product gene clusters from the environment: piecing environmental DNA gene clusters back together with TAR. *Biopolymers* **2010**, *93* (9), 833-844.
(3) Kallifidas, D.; Brady, S. F. Reassembly of functionally intact environmental DNA-derived biosynthetic gene clusters. *Methods Enzymol* **2012**, *517*, 225-239.
(4) Kim, S. H.; Lu, W.; Ahmadi, M. K.; Montiel, D.; Ternei, M. A.; Brady, S. F. Atolypenes, Tricyclic Bacterial Sesterterpenes Discovered Using a Multiplexed In Vitro Cas9-TAR Gene Cluster Refactoring Approach. *ACS Synth Biol* **2019**, *8* (1), 109-118.
(5) Frey, S.; Gorlich, D. A new set of highly efficient, tag-cleaving proteases for purifying recombinant proteins. *J Chromatogr A* **2014**, *1337*, 95-105.
(6) Brachmann, C. B.; Davies, A.; Cost, G. J.; Caputo, E.; Li, J.; Hieter, P.; Boeke, J. D. Designer deletion strains derived from Saccharomyces cerevisiae S288C: a useful set of strains and plasmids for PCR-mediated gene disruption and other applications. *Yeast* **1998**, *14* (2), 115-132.
(7) Wu, C.; Shang, Z.; Lemetre, C.; Ternei, M. A.; Brady, S. F. Cadasides, Calcium-Dependent Acidic Lipopeptides from the Soil Metagenome That Are Active against Multidrug-Resistant Bacteria. *J Am Chem Soc* **2019**, *141* (9), 3910-3919.
(8) Culp, E. J.; Sychantha, D.; Hobson, C.; Pawlowski, A. C.; Prehna, G.; Wright, G. D. ClpP inhibitors are produced by a widespread family of bacterial gene clusters. *Nat Microbiol* **2021**.
